# Polyribonucleotide nucleotidyltransferase 1 participates in metabolic-associated fatty liver disease pathogenesis by affecting lipid metabolism and mitochondrial homeostasis

**DOI:** 10.1016/j.molmet.2024.102022

**Published:** 2024-08-31

**Authors:** Canghai Guan, Xinlei Zou, Chengru Yang, Wujiang Shi, Jianjun Gao, Yifei Ge, Zhaoqiang Xu, Shaowu Bi, Xiangyu Zhong

**Affiliations:** 1 General Surgery Department, The 2nd Affiliated Hospital of Harbin Medical University, 148 Baojian Street, Harbin 150086, Heilongjiang Province, China; 2 The Key Laboratory of Myocardial Ischemia, Harbin Medical University, Ministry of Education, 148 Baojian Street, Harbin 150086, Heilongjiang, China

**Keywords:** Metabolic-associated fatty liver disease, PNPT1, Lipid metabolism, Mitochondrial membrane permeability, Mcl-1, PPARα

## Abstract

**Objective:**

Metabolic-associated fatty liver disease (MAFLD) represents one of the most prevalent chronic liver conditions worldwide, but its precise pathogenesis remains unclear. This research endeavors to elucidate the involvement and molecular mechanisms of polyribonucleotide nucleotidyltransferase 1 (PNPT1) in the progression of MAFLD.

**Methods:**

The study employed western blot and qRT-PCR to evaluate PNPT1 levels in liver specimens from individuals diagnosed with MAFLD and in mouse models subjected to a high-fat diet. Cellular studies investigated the effects of PNPT1 on lipid metabolism, apoptosis, and mitochondrial stability in hepatocytes. Immunofluorescence was utilized to track the subcellular movement of PNPT1 under high lipid conditions. RNA immunoprecipitation and functional assays were conducted to identify interactions between PNPT1 and Mcl-1 mRNA. The role of PPARα as an upstream transcriptional regulator of PNPT1 was investigated. Recombinant adenoviral vectors were utilized to modulate PNPT1 expression *in vivo*.

**Results:**

PNPT1 was found to be markedly reduced in liver tissues from MAFLD patients and HFD mice. *In vitro*, PNPT1 directly regulated hepatic lipid metabolism, apoptosis, and mitochondrial stability. Under conditions of elevated lipids, PNPT1 relocated from mitochondria to cytoplasm, modifying its physiological functions. RNA immunoprecipitation revealed that the KH and S1 domains of PNPT1 bind to and degrade Mcl-1 mRNA, which in turn affects mitochondrial permeability. The transcriptional regulator PPARα was identified as a significant influencer of PNPT1, impacting both its expression and subsequent cellular functions. Alterations in PNPT1 expression were directly correlated with the progression of MAFLD in mice.

**Conclusions:**

The study confirms the pivotal function of PNPT1 in the development of MAFLD through its interactions with Mcl-1 and its regulatory effects on lipid metabolism and mitochondrial stability. These insights highlight the intricate association between PNPT1 and MAFLD, shedding light on its molecular pathways and presenting a potential new therapeutic avenue for MAFLD management.

## Highlights

• PNPT1 expression is significantly downregulated in liver from MAFLD patients and HFD mouse models.
• PNPT1 regulates hepatic lipid metabolism, apoptosis, and mitochondrial stability in hepatocytes.
• Under high lipid conditions, PNPT1 moves from mitochondria to cytoplasm, exacerbating MAFLD via Mcl-1 interaction.
• PPARα acts as an upstream transcriptional regulator of PNPT1, influencing its role in MAFLD pathogenesis.

## Graphical abstract

**Figure:**
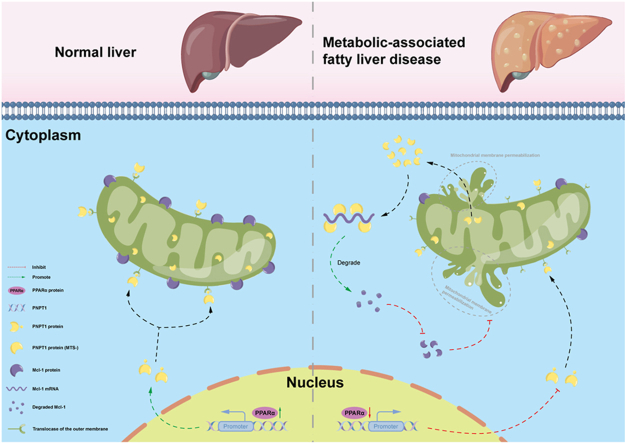

## 1 Introduction

Metabolic-associated fatty liver disease (MAFLD) ranks among the most prevalent chronic liver conditions globally, with a continually rising incidence and prevalence [[1], [2], [3], [4], [5]]. Research indicates that the prevalence of MAFLD in adults ranges from 20 to 30% in developed nations, with developing countries approaching similar rates [[6], [7], [8], [9], [10], [11], [12]]. MAFLD not only poses significant risks to liver health but is also closely associated with systemic diseases such as cardiovascular disorders and diabetes [[13], [14], [15], [16], [17], [18], [19]]. As a metabolic liver disorder, the pathogenesis of MAFLD involves irregularities in lipid metabolism, oxidative stress, inflammatory responses, and hepatocyte apoptosis [[20], [21], [22], [23]]. MAFLD encompasses two primary stages: simple fatty liver and metabolic dysfunction-associated steatohepatitis (MASH). Patients with simple fatty liver exhibit only hepatic fat accumulation, whereas those with MASH experience hepatocellular injury, an inflammatory response, and may ultimately progress to liver fibrosis, cirrhosis, and potentially hepatocellular carcinoma (HCC), highlighting the urgent need to understand its underlying mechanisms [24,25]. Despite this, the precise molecular mechanisms driving MAFLD development remain elusive. Thus, identifying key causal molecules and signaling pathways in MAFLD is crucial to elucidate its pathogenesis and devise preventive strategies.

Polyribonucleotide nucleotidyltransferase 1 (PNPT1) functions as an exoribonuclease involved in various RNA metabolic processes [[26], [27], [28], [29]]. Extensive studies have emphasized PNPT1's significant role in biological processes, including cell proliferation, apoptosis, and differentiation [[30], [31], [32]]. Specifically, PNPT1 can localize to mitochondria and form a complex with SUV3 RNA helicase to maintain mitochondrial functional stability via degrading mitochondrial RNAs [[33], [34], [35]]. PNPT1 also facilitates the decay of numerous messenger RNAs and microRNAs [36,37]. Abnormal expression of PNPT1 has been reported in various diseases including cancer and cardiovascular disorders [38,39]. However, its role in lipid metabolism associated diseases, particularly MAFLD, remains largely unexplored.

The Bcl-2 family proteins are well known for their extensive involvement in the regulation of apoptosis [40]. Pro-apoptotic members, such as Bax and Bad, promote the opening of the mitochondrial permeability transition pore (mPTP), leading to mitochondrial dysfunction [41]. Conversely, anti-apoptotic members like Mcl-1 stabilize the mitochondrial membrane potential to protect mitochondrial integrity [42]. In many diseases, the downregulation of Mcl-1 expression results in increased mitochondrial permeability, leading to cell apoptosis [43,44]. Therefore, a deeper exploration of the upstream molecules regulating Mcl-1, which in turn affect mitochondrial permeability, is of significant importance in elucidating the pathogenesis of MAFLD.

Given its pivotal regulatory role in cellular stress responses, particularly in modulating intracellular RNA degradation and mitochondrial function [45], PNPT1 is hypothesized to play a crucial role in MAFLD. These functions are essential in the development of MAFLD, as the pathology involves abnormal lipid metabolism and mitochondrial damage [46,47]. Our study is the first to reveal a crucial role of PNPT1 in the pathogenesis of metabolic-associated fatty liver disease. We demonstrated that PNPT1 expression was downregulated in MAFLD patients and animal models, suggesting its involvement in disease progression. Furthermore, we provided novel evidence that PNPT1 translocated from mitochondria to cytoplasm under high lipid conditions, and can enhance mitochondrial permeability by degrading the anti-apoptotic factor Mcl-1 mRNA. This increase in mitochondrial permeability allows further release of PNPT1 into the cytosol, forming a positive feedback loop with Mcl-1 to jointly control hepatocyte apoptosis and mitochondrial stability. Our study not only identifies PNPT1 as a novel molecule implicated in MAFLD pathogenesis but also elucidates its multifaceted regulatory mechanisms. We hope that these findings will provide valuable insights and directions for future MAFLD research and therapy, to address this significant health challenge.

## 2 Methods

### 2.1 Clinical samples

Hepatic tissue specimens were obtained from patients afflicted with liver diseases, courtesy of The 2nd Affiliated Hospital of Harbin Medical University. The Ethics Committee granted approval for all specimen procurement protocols. The patient inclusion criteria encompassed: 1) age range of 18–65 years; 2) confirmed pathophysiological and clinical diagnosis of either non-alcoholic fatty liver or non-alcoholic steatohepatitis; 3) voluntary provision of informed consent. The exclusion criteria included: 1) simultaneous severe maladies of vital organs such as the heart, liver, or kidneys; 2) presence of malignant neoplasms; 3) pregnancy or lactation; 4) psychiatric disorders. A subset of patients consented to undergo percutaneous liver biopsy, and liver tissue specimens were procured in accordance with standard protocols. A fraction of these samples was utilized for pathological diagnosis, while the remaining tissue was immediately cryopreserved in liquid nitrogen. Stringent quality control measures were implemented on all procured samples to ensure their integrity and accuracy, preceding any subsequent experimental procedures. The general characteristics of the MAFLD human liver samples were listed in supplementary Table 1.

### 2.2 Cell culture and transfection

HepG2 and AML12 cell lines, obtained from the Cell Bank of the Chinese Academy of Sciences (Shanghai, China), were cultured in DMEM medium (Gibco, New York, USA), supplemented with 10% fetal bovine serum (Invitrogen, Carlsbad, USA), 100 U/ml penicillin, and 100 μg/ml streptomycin (Gibco), under conditions of 37 °C and 5% CO_2_. To replicate *in vitro* conditions of fatty liver, cells in the logarithmic growth phase were exposed to an equimolar concoction of oleic acid (OA, Sigma, St. Louis, MO) and palmitic acid (PA, Sigma) at an OA:PA ratio of 2:1 (final concentration 200 μM) for a duration of 24 h. For gene transfection experiments, HepG2 and AML12 cells were dispersed into 6-well plates at a density of 2 × 105 cells/well and incubated overnight. At approximately 70% confluence, transient transfection was carried out utilizing Lipofectamine 2000 (Invitrogen) in accordance with the manufacturer's guidelines. Constructs or siRNAs (GenePharma, Shanghai, China) were gently amalgamated and incubated for 6 h prior to replacement with complete medium for continued incubation. Cells were harvested 24–48 h post-transfection for subsequent assays. Each experiment included blank and negative controls, and all samples were executed in triplicate.

### 2.3 Quantitative real-time PCR (qRT-PCR)

RNA Extraction: Total RNA extraction from cells and tissues was accomplished using Trizol reagent (Invitrogen), adhering to the manufacturer's guidelines. RNA concentration and purity were scrutinized via UV spectrophotometry, accepting A260/A280 ratios within the 1.8–2.0 range. Two micrograms of RNA was reverse transcribed into cDNA employing a commercially available kit (Roche, Penzberg, Germany). qRT-PCR was carried out employing the SYBR Green method (Roche) on a C1000 Thermal Cycler (Bio-Rad, Hercules, USA). Primer sequences are delineated in supplementary Table 2. Each 20 μl reaction encompassed 10 μl SYBR Green, 1 μl of both forward and reverse primers (10 μM), 2 μl template cDNA, and nuclease-free water. GAPDH and β-actin functioned as internal references. Triplicate wells were arranged for each sample, and experiments were repeated at least three times. The 2-ΔΔCT method was utilized for the calculation of relative expression.

### 2.4 Western blot

Total cellular protein was extracted using RIPA lysis buffer (Beyotime, Beijing, China) and quantified via the BCA method. 50 μg protein samples were subjected to SDS-PAGE and transferred onto PVDF membranes. Membranes were blocked with 5% skim milk at ambient temperature for 1 h, followed by overnight incubation with primary antibodies at 4 °C. Subsequent to TBST washes, appropriate secondary antibodies were applied at room temperature for 1 h. Chemiluminescence detection was conducted using ECL reagents (Beyotime). Band densities were analyzed using Image J software, with target protein levels normalized to the internal control Tubulin. Each group comprised triplicate wells, and experiments were replicated at least three times. Antibodies utilized are listed in supplementary Table 3.

### 2.5 Lipid accumulation detection

Cells or tissues fixed with 4% paraformaldehyde underwent dehydration with 60% isopropanol, followed by staining with Oil red O working solution (Solarbio, Beijing, China) for 15 min, in compliance with the manufacturer's protocol. This was succeeded by swift washes with 60% isopropanol 2–3 times, washing with water, and mounting. Images were captured under a microscope.

In the case of Nile red staining, cells were washed with PBS and incubated with Nile red staining solution (Solarbio) for 10 min. Following a PBS wash, fluorescence microscopy was employed to observe the stained cells.

Triglyceride (TG) and total cholesterol (TC) levels were measured utilizing chemiluminescent enzyme-linked immunosorbent assays kits (Nanjing Jiancheng Bioengineering Institute, Nanjing, China). In accordance with the manufacturer's guidance, TG and TC contents were detected in cell lysate or mouse serum. Triplicate wells were delineated for each sample, and experiments were replicated at least three times. Optical density values were recorded and averaged. All procedures strictly adhered to the prescribed manuals.

### 2.6 TUNEL staining

The TUNEL cell apoptosis detection kit (Roche) was utilized according to the instructions provided. Treated cells were fixed on polylysine-coated slides and incubated with the TUNEL reaction mixture for 1 h, followed by DAPI staining. Under fluorescence microscopy, the percentage of TUNEL-positive cells was determined to evaluate apoptosis. Triplicates were performed for each group, and experiments were repeated at least three times.

### 2.7 Mitochondrial membrane potential and permeability pore detection

Mitochondrial membrane potential was assessed using the JC-1 dye (Beyotime). Treated cells were incubated with JC-1 working solution for 20 min, washed, and observed under fluorescence microscopy. Healthy cells exhibited aggregated red fluorescence, while cells with decreased mitochondrial membrane potential showed green fluorescence. The ratio of red to green fluorescence intensity was calculated by image analysis to evaluate mitochondrial membrane potential levels.

The mitochondrial permeability transition pore (mPTP) opening was detected using the Mitochondrial Permeability Transition Pore Assays Kit (Beyotime). Cells were initially stained with calcein-AM to yield cytoplasmic green fluorescence, followed by treatment with CoCl2 to quench fluorescence. Cytoplasmic fluorescence leakage was monitored as an indicator of mPTP opening.

### 2.8 Transmission electron microscopy

Cell samples were pre-fixed with 2.5% glutaraldehyde (Sigma) for 2 h, washed with 0.1M phosphate buffer (pH 7.4, Solarbio), and post-fixed with 1% osmium tetroxide (Sigma) for 1.5 h. Following dehydration with ethanol and infiltration with acetone butyl ester (Sigma), samples were embedded, sectioned, and double stained with uranyl acetate and lead citrate (Sigma). Mitochondrial morphology was observed under a JEOL-1230 transmission electron microscope (JEOL, Tokyo, Japan).

### 2.9 Immunofluorescence staining

Standard immunofluorescence staining was undertaken to examine protein localization. Cells on slides were fixed with 4% paraformaldehyde for 15 min, permeabilized with 0.2% Triton X-100 (Solarbio) at room temperature for 15 min, blocked with 10% goat serum (Solarbio) at room temperature for 30 min, incubated with primary antibody at 4 °C overnight, and fluorescent secondary antibody at room temperature for 1 h. Nuclei were counterstained with DAPI for 5 min. Images were captured under a laser scanning confocal microscope (Leica) in fluorescence mode.

### 2.10 Mitochondrial isolation

Following the mitochondrial isolation kit protocol (Beyotime), mitochondria were isolated from cells by differential centrifugation. The enrichment of the PNPT1 protein in the mitochondria was evaluated using Western blotting, with COX IV and Tubulin used as markers for mitochondria and the cytoplasm, respectively.

### 2.11 RNA immunoprecipitation (RIP)

The RIP assays kit protocol (Millipore) was utilized. Cell lysates were prepared, with one portion preserved as an input control. The target antibody or control IgG was added to the remaining lysate and incubated overnight at 4 °C to allow the antibody to bind to endogenous protein complexes. The Protein A/G beads were washed, added to the samples, and incubated for 2 h at 4 °C to bind protein complexes via the antibody Fc regions. The beads were precipitated and the supernatant discarded. The beads were washed with RIPA buffer. The target proteins were eluted by boiling in a water bath and analyzed by Western blotting. The relative levels were calculated by comparing to the input control to evaluate protein interactions.

### 2.12 Actinomycin D (Act D) assays

Act D (MedChemExpress, New Jersey, USA) at a concentration of 5 μg/ml was used to examine the effect of PNPT1 on the stability of Mcl-1 mRNA. Transfected cells were treated to inhibit new mRNA synthesis. At specified time points (0, 4, 8, 12 h), cells were collected for total RNA extraction. Mcl-1 mRNA levels were measured by qRT-PCR and the relative expression at each time point was calculated. The degradation rate of Mcl-1 mRNA was monitored to determine the impact of PNPT1 on its stability.

### 2.13 Chromatin immunoprecipitation (ChIP)

The ChIP kit protocol (Millipore) was followed. When cells reached 90% confluence, they were fixed with 1% formaldehyde for 10 min. Glycine (Solarbio) was added to stop the reaction. After ultrasonic lysis, cell lysates were incubated with control IgG or the target antibody overnight at 4 °C to form antibody-DNA-protein complexes. Pre-washed protein A/G beads were then added and incubated for 2 h. The immunoprecipitates were isolated using a magnetic rack and washed with 0.1% SDS to remove non-specific DNA. Proteins were digested with proteinase K and the DNA was extracted with chloroform. The target gene sequences were PCR amplified and the enrichment of ChIP DNA over the input control was calculated to evaluate transcription factor-DNA binding.

### 2.14 Dual luciferase reporter assays

A luciferase reporter constructs and an internal control Renilla plasmid were co-transfected into cells using Lipofectamine. After 48 h, cells were lysed and the Renilla luciferase activity was first measured as an internal reference. Following this, a luciferase buffer was added to assess reporter activity in accordance with the kit protocol (Promega, Wisconsin, USA). The fluorescence signals generated by the enzymatic reactions were used to analyze changes in reporter transcription. Experiments were conducted in triplicate. The reporter activity was normalized to the internal control for each sample, and the relative activity was calculated for each group.

### 2.15 Animal models

Eight-week-old male C57BL/6 mice were fed either normal diet (ND) or high-fat diet (HFD) for 8 weeks to induce MAFLD (n = 7 per group). For overexpression, AAV8-PNPT1 was injected via the tail vein using vector GV704 ApoE/hAATp-MCS-SV40 PolyA (Genechem, Shanghai, China). For knockdown, AAV8-shPNPT1 with vector GV698 pAAV-ApoE/hAATp-EGFP-MIR155(MCS)-SV40 PolyA (Genechem) was used. Serum and tissues were collected after 8 weeks. All animal experiments complied with ethical regulations under strict controls.

### 2.16 Tissue sectioning and staining

Liver tissues were rapidly frozen in OCT (Sakura Finetek, Torrance, USA). Ten micrometer cryosections were cut using a cryostat (Leica) and stained with Oil red O solution for 15 min, followed by 60% isopropanol wash. Lipid droplets were observed under a microscope. Other tissue samples were fixed in 10% neutral formalin, dehydrated in graded ethanol, cleared in xylene, and embedded in paraffin. Four micrometer sections were cut using a microtome (Leica) and processed for H&E staining (Solarbio) to examine morphology, Sirius red staining (Solarbio) for collagen fibers, and Masson's trichrome staining (Solarbio) to evaluate collagen fibrosis.

### 2.17 Statistical analysis

All statistical analyses were performed using the SPSS software (IBM SPSS, New York, USA). Graphs were generated with GraphPad Prism 8 (GraphPad Software, La Jolla, USA). Quantitative data are expressed as mean ± SD. Comparisons between two groups were conducted by unpaired Student's *t*-test, while one-way ANOVA was used for multiple groups. Correlations were determined by the Pearson coefficient. A p-value of <0.05 was considered statistically significant.

## 3 Results

### 3.1 PNPT1 is downregulated in MAFLD and MASH tissues and hyperlipid-induced cells

**Figure 1:**
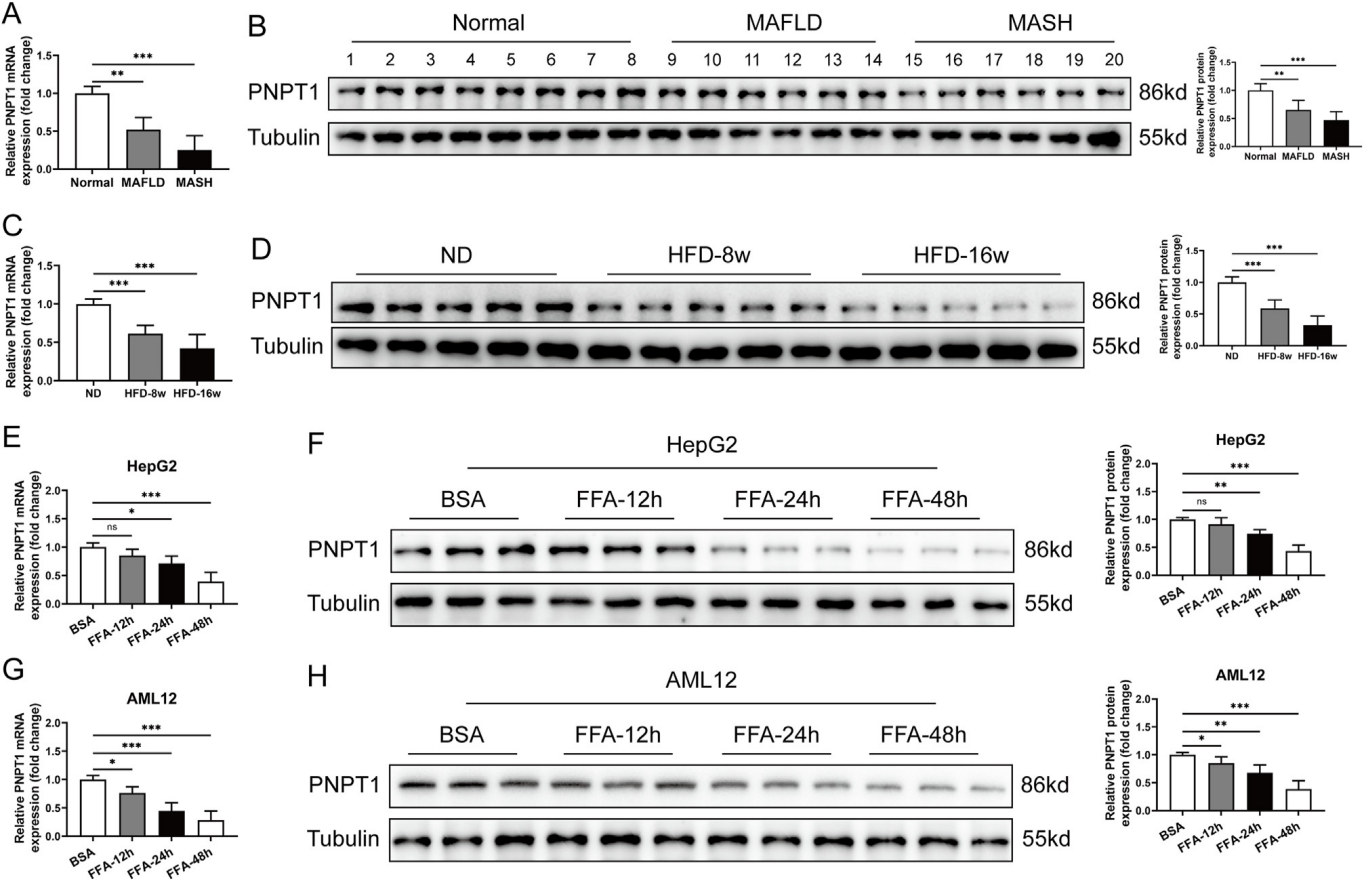
**PNPT1 expression in MAFLD and HFD conditions**. (A, B) qRT-PCR and western blot analysis showing significant downregulation of PNPT1 in human MAFLD (*n* = 6) and MASH (*n* = 6) tissues compared to normal liver tissues (*n* = 8). (C, D) qRT-PCR and western blot results confirming lower levels of PNPT1 protein in HFD (8w and 16w) mice compared to ND mice (*n* = 5). (E, F) qRT-PCR and western blot showing PNPT1 protein reduction in HepG2 cells post FFA treatment (*n* = 6). (G, H) Decreased PNPT1 mRNA and protein levels in AML12 cells after FFA induction, as evidenced by qRT-PCR and western blot (*n* = 6). ∗*P* < 0.05, ∗∗*P* < 0.01.

In this study, we initially investigated the expression of PNPT1 in tissues from patients with MAFLD and MASH, as well as in HFD induced mouse model. Through qRT-PCR and western blotting techniques, we discovered that PNPT1 was significantly downregulated in the tissues of MAFLD patients compared to normal liver tissues (Figure 1A,B). Similarly, the expression of PNPT1 decreased in the HFD mouse model, and the decline was more pronounced with the progression of MAFLD (Figure 1C,D). We also treated HepG2 and mouse AML12 hepatocytes *in vitro* with high lipids using FFA (palmitic acid + oleic acid), and PNPT1 mRNA and protein levels of both hepatocytes decreased with increasing treatment time compared to BSA normal controls (Figure 1E–H).

### 3.2 PNPT1 can regulate the cellular lipid metabolism

**Figure 2:**
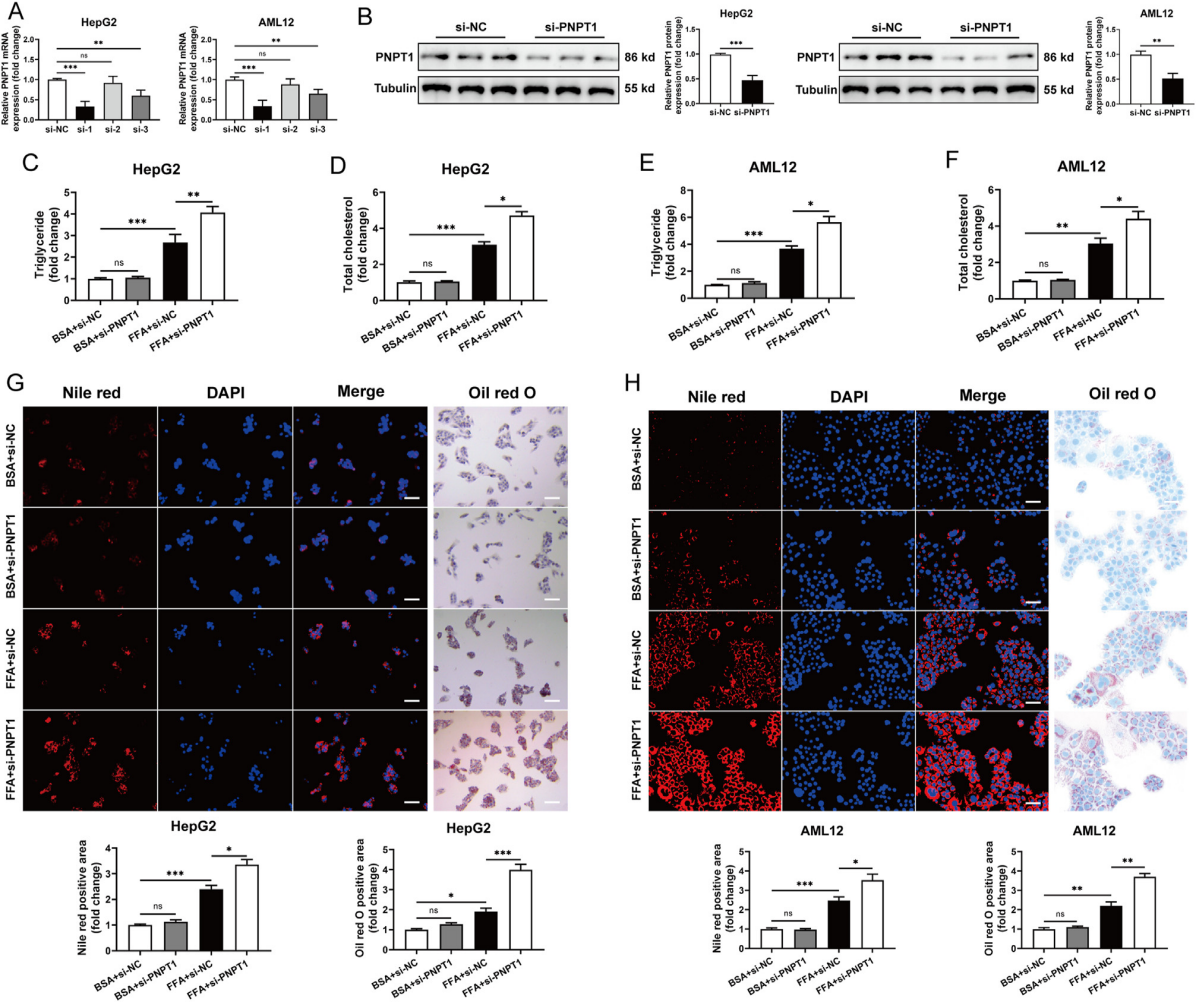
**PNPT1 modulation effects on lipid metabolism in liver cells**. (A, B) Efficacy of PNPT1 silencing verified by qRT-PCR and western blot in HepG2 and AML12 cells (*n* = 5). (C-F) Quantification of TG and TC levels showing increased and decreased lipid accumulation in FFA-induced cells upon PNPT1 silencing (*n* = 5). (G, H) Nile red and Oil red O staining with corresponding quantifications demonstrating enhanced lipid droplet accumulation in PNPT1 knockdown HepG2 and AML12 cells (FFA-treated) (*n* = 5). ∗*P* < 0.05, ∗∗*P* < 0.01, ∗∗∗*P* < 0.001.

We proceeded to explore the role of PNPT1 in cellular lipid metabolism. By transfecting HepG2 and AML12 cells with PNPT1 siRNA or overexpression plasmids, we induced significant downregulation or upregulation of PNPT1 (Figure 2A,B and Figure S1A, B). Measurements of TG and TC confirmed elevated levels of both in FFA-induced cells following PNPT1 silencing (Figure 2C–F). Additionally, observations with Oil red O and Nile red staining further revealed increased numbers and sizes of lipid droplets in FFA-treated cells after PNPT1 knockdown (Figure 2G,H). Conversely, overexpression of PNPT1 resulted in reduced formation of lipid droplets and decreased levels of triglycerides and cholesterol in FFA-induced cells (Figure S1C-H). These findings collectively indicate that PNPT1 significantly influences cellular lipid metabolism.

### 3.3 PNPT1 regulate cell apoptosis and mitochondrial permeability

**Figure 3:**
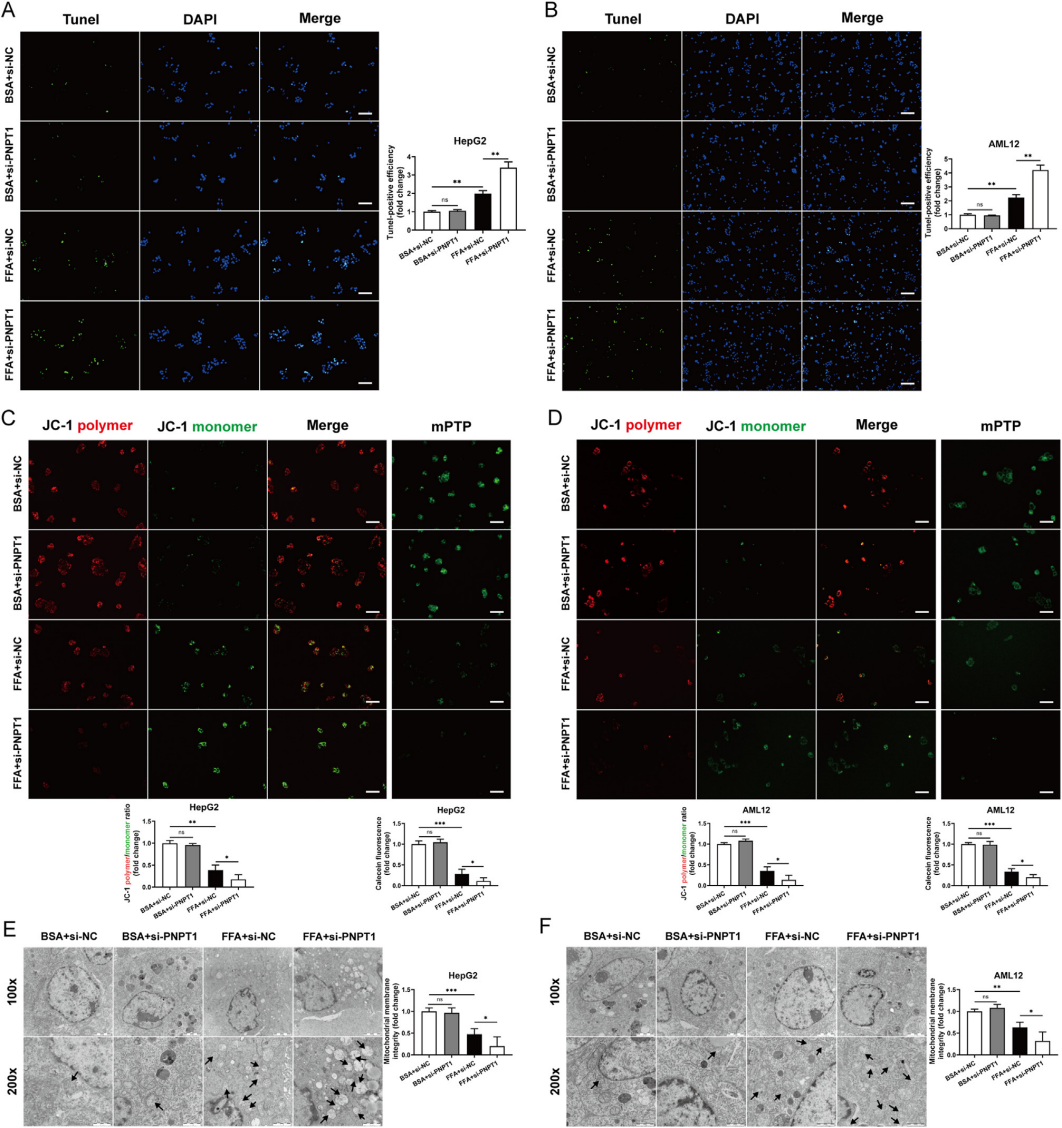
**Impact of PNPT1 on apoptosis and mitochondrial integrity in** HepG2 and AML12 **cells**. (A, B) Tunel assays indicating a significant increase in apoptosis rates in FFA-treated HepG2 and AML12 cells following PNPT1 silencing (*n* = 5). (C, D) JC-1 staining and mPTP assays showing changes in mitochondrial membrane potential and increased permeability pore opening in cells treated with FFA and si-PNPT1 (*n* = 5). (E, F) Transmission electron microscopy images illustrating increased mitochondrial swelling and deformation in FFA-treated cells after PNPT1 knockdown (*n* = 3). ∗*P* < 0.05, ∗∗*P* < 0.01, ∗∗∗*P* < 0.001.

TUNEL assays showed a significant increase in apoptosis rates in FFA-treated cells following PNPT1 siRNA transfection (Figure 3A,B), whereas overexpression of PNPT1 decreased apoptosis in liver cells (Figure S2A, B). Previous research has demonstrated that PNPT1 localizes to the intermembrane space of mitochondria and helps to maintain mitochondrial stability [48,49]. Therefore, we postulated that PNPT1 might modulate apoptosis by influencing mitochondrial homeostasis. Using JC-1 staining and mPTP assays, we observed alterations in mitochondrial membrane potential and increased permeability pore opening after FFA treatment, which were exacerbated by PNPT1 silencing but ameliorated by overexpression (Figure 3C,D and Figure. S2C, D). Moreover, electron microscopy revealed increased mitochondrial swelling and deformation in FFA-induced cells due to PNPT1 knockdown (Figure 3E,F), while overexpression mitigated these changes (Figure S2E, F). This data confirms the critical role of PNPT1 in regulating apoptosis and maintaining mitochondrial stability.

### 3.4 High lipid induction causes PNPT1 translocation to cytoplasm and exacerbates apoptosis

**Figure 4:**
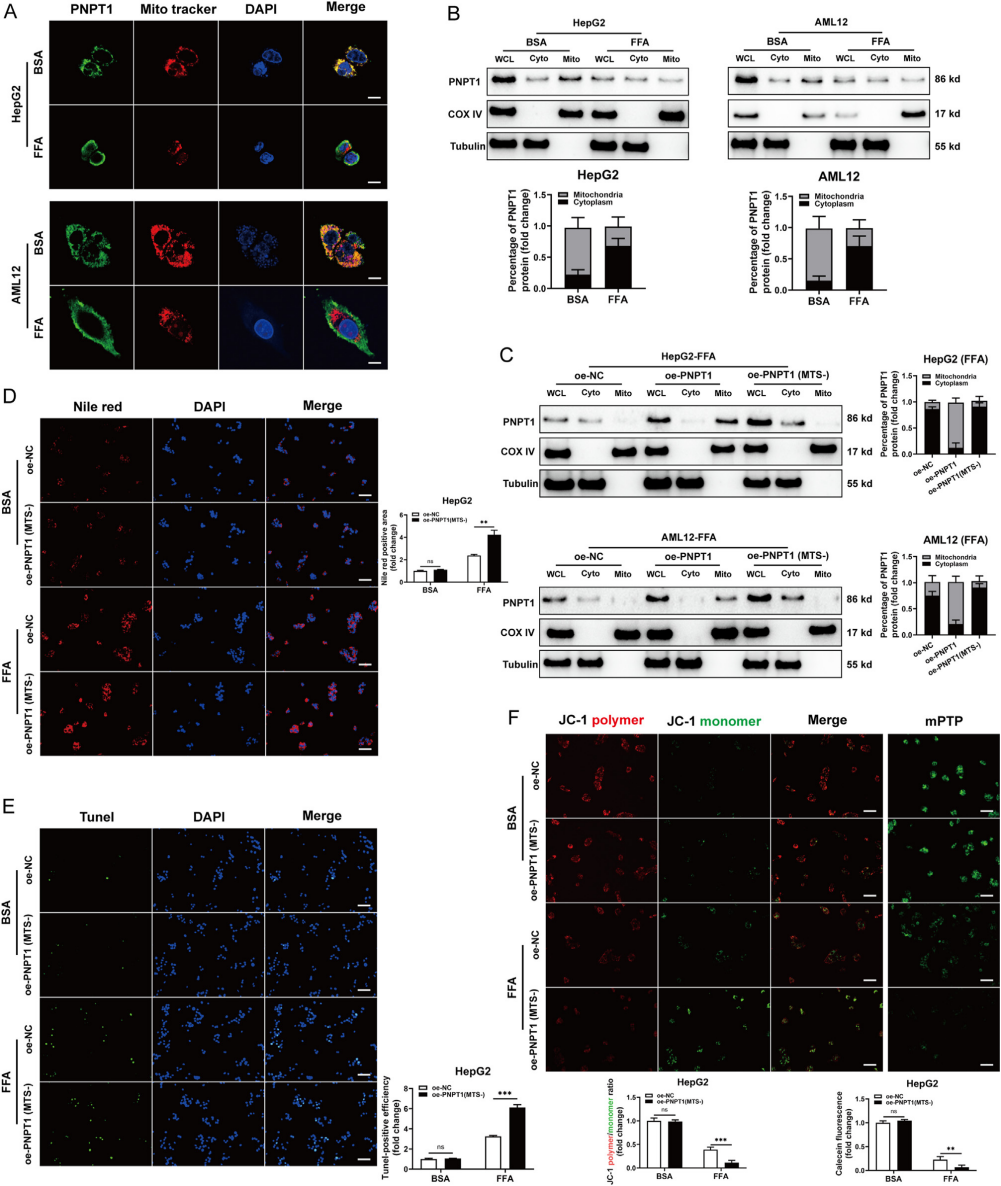
**Subcellular localization of PNPT1 and its consequences on apoptosis and mitochondrial integrity under high lipid conditions**. (A) Immunofluorescence images showing PNPT1 localization in HepG2 and AML12 cells. Under control conditions (BSA), PNPT1 is localized in mitochondria, while FFA treatment leads to its enrichment in the cytoplasm (*n* = 3). (B) Western blot analysis of whole cell (WCL), cytoplasm (Cyto), and mitochondrial (Mito) fractions from HepG2 and AML12 cells, indicating decreased PNPT1 in WCL and Mito but increased in Cyto upon FFA treatment (*n* = 3). (C) Western blot analysis showing the expression of PNPT1 in liver cells transfected with either normal PNPT1 overexpression construct (oe-PNPT1) or MTS-truncated PNPT1 (oe-PNPT1(MTS-)), demonstrating specific increase in cytoplasmic PNPT1 without affecting in mitochondrial by MTS truncation (*n* = 3). (D) Nile red staining indicating heavier lipid accumulation in oe-PNPT1(MTS-) cells than oe-NC in FFA-induced HepG2 (*n* = 5). (E) Tunel assays revealing FFA-induced cell apoptosis was enhanced after overexpressing the MTS-truncated PNPT1 (*n* = 5). (F) JC-1 staining and mPTP assays showed that oe-PNPT1(MTS-) promoted the loss of mitochondrial membrane potential of FFA-treated cells and enhanced the opening of mPTP (*n* = 5). ∗∗*P*<0.01.

We further investigated the subcellular localization of PNPT1 under conditions of high lipid content. Typically localized in the mitochondria, PNPT1 was found primarily in the mitochondria in control cells, but significantly enriched in the cytoplasm in cells treated with high lipids (Figure 4A), possibly due to increased mitochondrial permeability induced by FFA. This was confirmed by analyzing PNPT1 levels in whole cell, cytoplasmic, and isolated mitochondrial fractions, where FFA induction reduced whole cell and mitochondrial PNPT1 expression but increased cytoplasmic levels (Figure 4B). This confirmed the translocation of PNPT1 from mitochondria to cytoplasm.

Considering this significant observation, we investigated whether increased cytoplasmic PNPT1 played a regulatory role in FFA-treated hepatocytes. Previous findings had demonstrated that mitochondrial PNPT1 enrichment could enhance stability and reduce apoptosis and lipid accumulation. As the N-terminus of PNPT1 contains a mitochondrial targeting sequence (MTS), we generated an MTS-truncated PNPT1 overexpression construct (oe-PNPT1(MTS-)) to specifically increase cytoplasmic levels. As anticipated, oe-PNPT1 increased mitochondrial and whole cell PNPT1, while oe-PNPT1(MTS-) specifically increased whole cell and cytoplasmic PNPT1 without affecting mitochondrial levels (Figure 4C).

Transfection with oe-PNPT1(MTS-) resulted in increased lipid accumulation, apoptosis, and mitochondrial permeability in FFA-treated cells, contrasting with oe-PNPT1. This suggests that post-FFA induction, increased cytoplasmic PNPT1 disrupts mitochondrial stability, thereby enhancing apoptosis and metabolic defects (Figure 4D–F and Figure S3A-C). These findings reveal that PNPT1 undergoes subcellular redistribution under high lipid conditions, and increased cytoplasmic PNPT1 negatively impacts mitochondrial stability and apoptosis.

### 3.5 Cytoplasmic PNPT1 promotes Mcl-1 degradation

**Figure 5:**
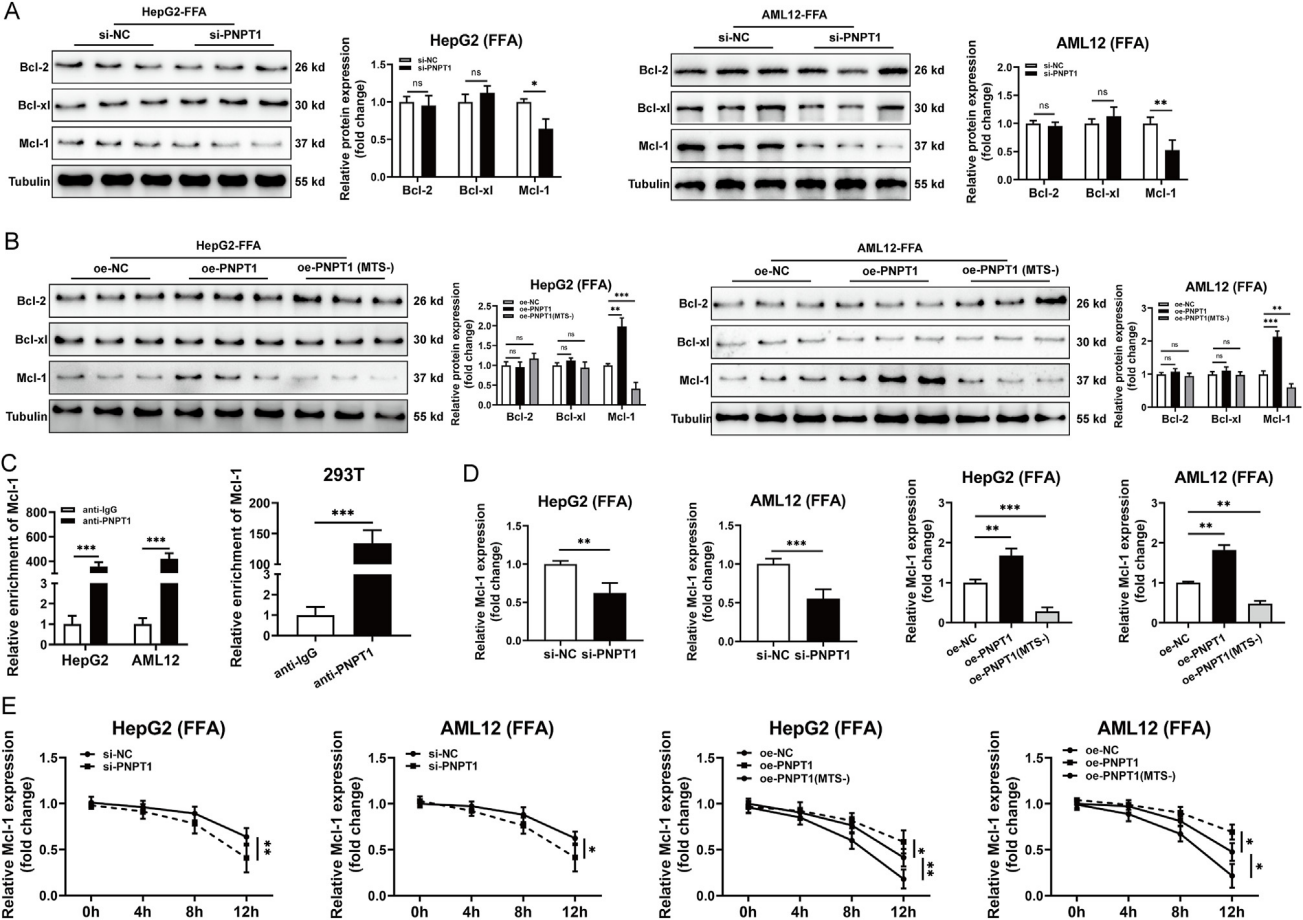
**The role of cytoplasmic PNPT1 in Mcl-1 degradation and mitochondrial permeability regulation**. (A) Western blot analysis showing the levels of Bcl-2 family proteins in HepG2 and AML12 cells following PNPT1 knockdown. There is no significant change in Bcl-2 and Bcl-xl, but an observable decrease in Mcl-1 levels (*n* = 3). (B) Comparing the effects of full-length and MTS-truncated PNPT1 overexpression on Mcl-1 expression after FFA induction, with Mcl-1 showing a decrease when cytoplasmic PNPT1 is increased (*n* = 3). (C) RIP assays demonstrating the binding of PNPT1 to Mcl-1 mRNA (*n* = 5). (D) The expression of Mcl-1 mRNA increased and decreased post-PNPT1 knockdown and MTS-truncated PNPT1 overexpression, respectively (*n*=5). (E) Act D assays results showing differences in Mcl-1 mRNA stability under conditions of PNPT1 knockdown and overexpression of either full-length or MTS-truncated PNPT1 (*n* = 5). ∗*P* < 0.05, ∗∗*P* < 0.01, ∗∗∗*P* < 0.001.

Further investigation revealed that cytoplasmic PNPT1, upon FFA treatment, exacerbates mitochondrial permeability. Since mitochondrial permeability is controlled by Bcl-2 family proteins [41,44], we hypothesized that cytoplasmic PNPT1 might influence this permeability by modulating these proteins. Western blotting revealed no change in Bcl-2 and Bcl-xl, but a decrease in Mcl-1 following PNPT1 knockdown (Figure 5A). However, compared with overexpression of the MTS-truncated PNPT1, Mcl-1 increased with the overexpression of full-length PNPT1 (Figure 5B), indicating that cytoplasmic PNPT1 could influence permeability by regulating Mcl-1.

As a ribonuclease, cytoplasmic PNPT1 is capable of promoting mRNA degradation. We postulated that translocated PNPT1 might degrade Mcl-1 mRNAs to modulate their expression. RIP experiments revealed that PNPT1 observably bound to Mcl-1 mRNA (Figure 5C). Additionally, Mcl-1 mRNA increased with full-length PNPT1 but decreased with the overexpression of MTS-truncated PNPT1 or PNPT1 knockdown (Figure 5D). Actinomycin D tests demonstrated that Mcl-1 mRNA degradation was enhanced by MTS-truncated but reduced by full-length PNPT1 overexpression (Figure 5E). In conclusion, cytoplasmic PNPT1, upon FFA treatment, may regulate Mcl-1 expression by degrading its mRNA, revealing a role for PNPT1 in modulating mitochondrial permeability and the expression of Bcl-2 family proteins.

### 3.6 PNPT1 binds Mcl-1 and forms a feedback loop to regulate apoptosis and mitochondrial stability

**Figure 6:**
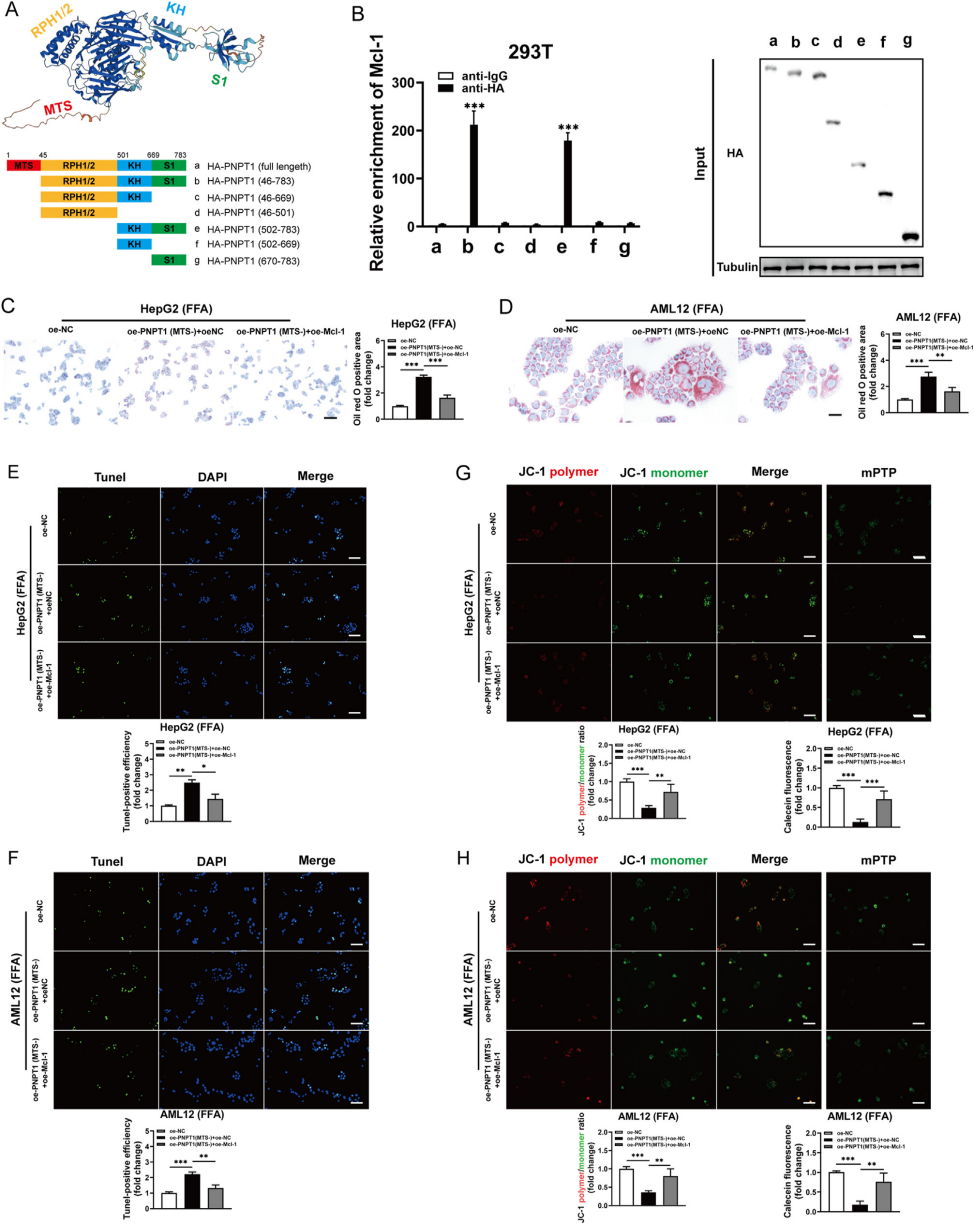
**Interaction of PNPT1 with Mcl-1 mRNA and its impact on apoptosis and mitochondrial stability**. (A) Schematic representation of PNPT1 protein with various domains and HA-tagged truncation constructs used in the study. (B) RIP analysis identifying the N-terminal region (amino acids 502-783) containing the KH and S1 domains of PNPT1 as critical for binding to Mcl-1 mRNA (*n* = 5). (C, D) The Oil red O staining showed that overexpression of Mcl-1 in FFA-induced hepatocytes reversed the increase in lipid accumulation observed in MTS-truncated PNPT1 overexpression. (E, F) Tunel staining reveals apoptotic cell counts in HepG2 and AML12 (FFA-treated) with altered PNPT1 expression and subsequent Mcl-1 overexpression. (G, H) JC-1 staining depicts mitochondrial membrane potential changes, and concurrent mPTP assays results show pore opening in cells treated with FFA, MTS-truncated PNPT1, and Mcl-1 overexpression (*n* = 5). ∗*P* < 0.05, ∗∗*P* < 0.01, ∗∗∗*P* < 0.001.

To determine the essential binding site of PNPT1 in Mcl-1 mRNA degradation, we created truncation constructs with N-terminal HA-tags (Figure 6A). RIP experiments indicated that the N-terminal region of PNPT1, specifically amino acids 502–783 containing the KH and S1 domains, bound to Mcl-1 (Figure 6B). In FFA-induced hepatocytes, the reduced lipid droplet production, the anti-apoptotic and mitochondrial protective effects after PNPT1 overexpression were reversed by silenced Mcl-1 or the Mcl-1 inhibitor S63845 (Figures. S4A-H and S5A-F), suggesting Mcl-1 mediates these effects. Conversely, overexpression of Mcl-1 rescued the reduced lipid droplet production and the pro-apoptotic and mitochondrial permeability increasing effects of MTS-truncated PNPT1 (Figure 6C–H). Collectively, these findings demonstrate that the N-terminal region, particularly the KH and S1 domains of PNPT1, is crucial for binding Mcl-1 mRNA. Cytoplasmic PNPT1 degrades Mcl-1, further releasing PNPT1 into the cytoplasm, ultimately forming a feedback loop with Mcl-1 to jointly regulate apoptosis and mitochondrial stability.

### 3.7 PPARα is an upstream transcription factor of PNPT1

**Figure 7:**
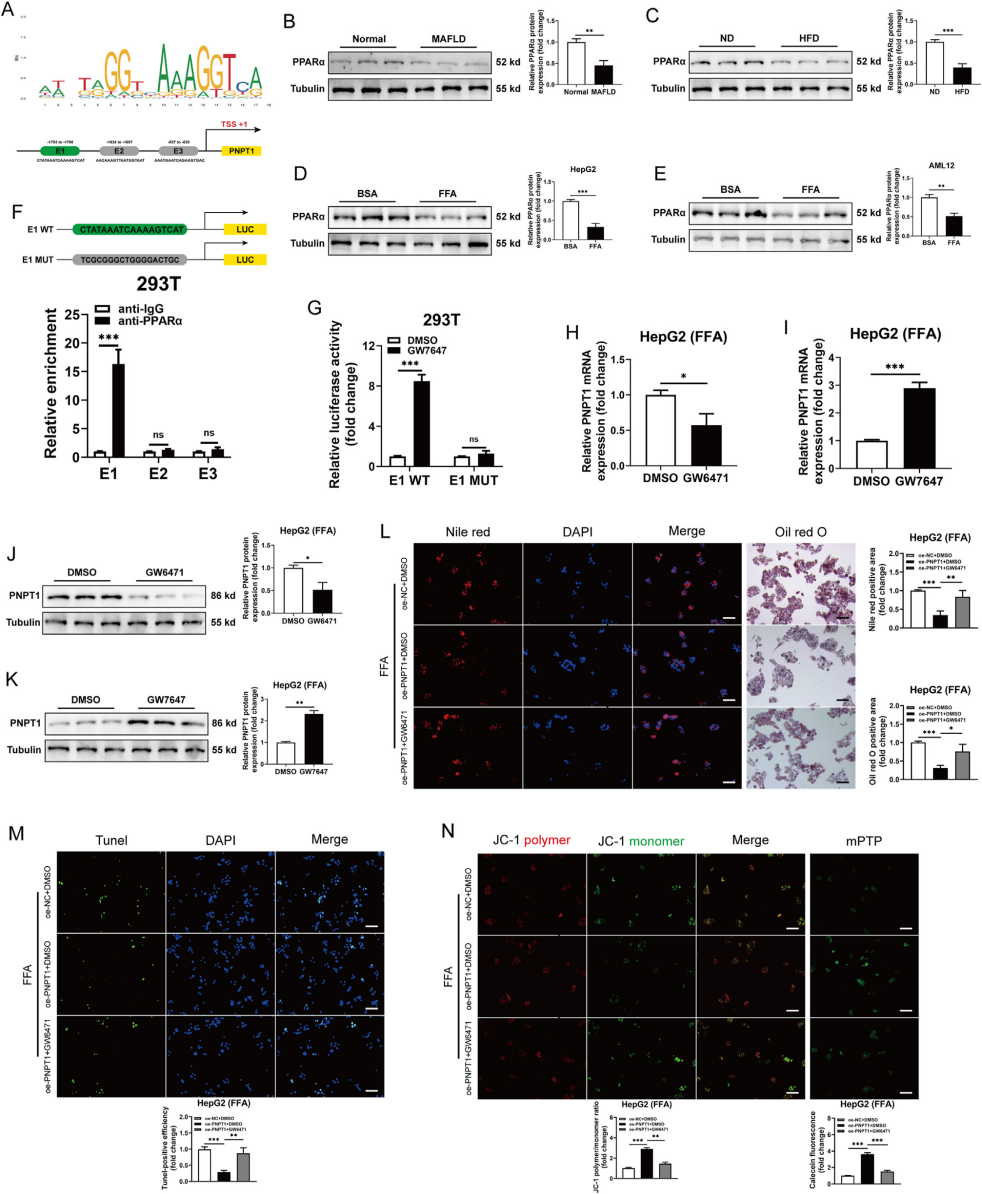
**PPARα regulation of PNPT1 expression and its effects on cellular lipid metabolism and mitochondrial stability**. (A) Diagram of the PNPT1 promoter with potential PPARα binding sites. (B-E) Western blot analysis showing PPARα levels in MAFLD tissues (*n* = 8), HFD mice (*n* = 6), and treated cells (*n* = 5), indicating downregulation in disease models. (F) ChIP assays confirmed that PPARα regulates PNPT1 mainly with binding E1 sites, compared with the predicted E2 and E3 binding sites (*n* = 5). (G) Luciferase reporter gene assay showed that PPARα agonist could significantly promote the fluorescence activity of wild-type (WT) E1 vector compared with E1 mutant (MUT) (*n* = 5). (H-K) PPARα antagonist (GW6471) reduces, while agonist (GW7647) increases, PNPT1 expression in HepG2 cells (FFA-treated), as shown by qRT-PCR and western blot (*n* = 5). (L-N) Nile red staining, Tunel, and JC-1/mPTP assays in HepG2 cells treated with FFA indicate that PNPT1 overexpression reduces lipid accumulation, apoptosis, and mitochondrial permeability, which are partially reversed by GW6471 (*n* = 5). ∗*P* < 0.05, ∗∗*P* < 0.01, ∗∗∗*P* < 0.001.

To investigate the upstream regulation of decreased PNPT1 expression in MAFLD, bioinformatic predictions revealed three potential PPARα binding sites (E1, E2, E3) in the PNPT1 promoter (Figure 7A). We confirmed that PPARα was downregulated in MAFLD tissues, HFD mice, and cells (Figure 7B–E). ChIP and luciferase reporter assays verified that PPARα primarily binds site E1, thereby regulating PNPT1 expression (Figure 7F,G).

In HepG2 and AML12 cells, the PPARα antagonist GW6471 decreased PNPT1 expression, while the agonist GW7647 increased it (Figures 7H–K and S6A-D). Rescue experiments indicated that the decreases in lipid accumulation, apoptosis, and mitochondrial permeability mediated by PNPT1 overexpression could be partially reversed by GW6471 following FFA treatment (Figure 7L-N). Conversely, GW7647 mitigated the effects of PNPT1 knockdown (Figure S6E-G). Thus, PPARα may modulate lipid metabolism and apoptosis through PNPT1, shedding light on the mechanisms of PNPT1 regulation.

### 3.8 *In vivo* PNPT1 regulation impacts MAFLD progression

**Figure 8:**
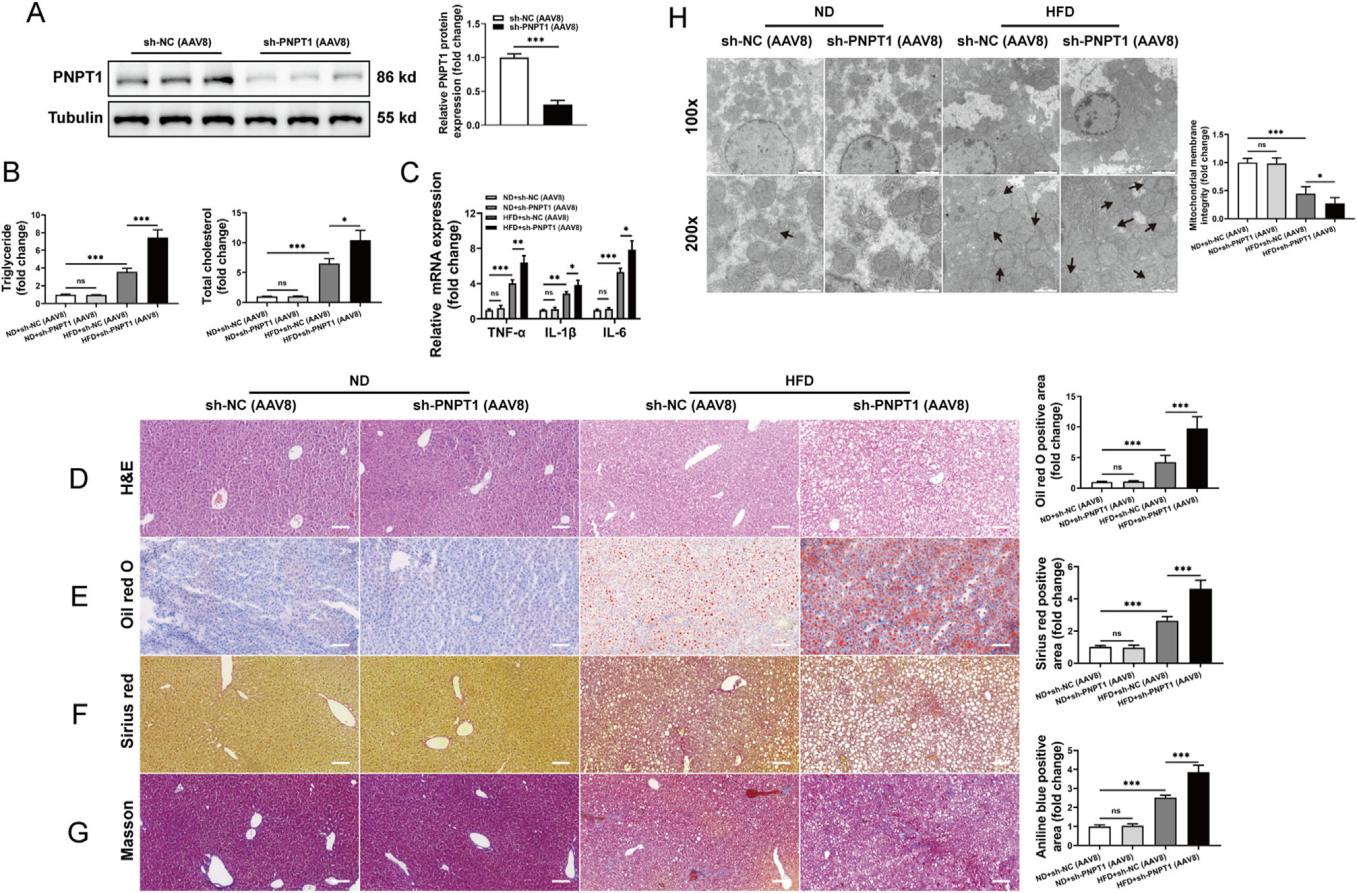
**Effects of PNPT1 knockdown on MAFLD progression *in vivo***. (A) Western blot confirmation of PNPT1 knockdown in mouse livers using AAV8 vectors (*n* = 6). (B) Quantitative analysis of hepatic TG and TC levels showing increases upon PNPT1 knockdown (*n* = 6). (C) In PNPT1-knockdown mouse liver, the expression of pro-inflammatory factors (TNF-α, IL-6, IL-1β) was significantly upregulated (*n* = 6). (D-G) Histological staining (H&E, Oil red O, Sirius red, and Masson's trichrome) of liver sections indicating increased lipid accumulation and fibrosis in PNPT1 knockdown mice compared to control (*n* = 6). (H) Transmission electron microscopy images revealing mitochondrial swelling in mouse livers with reduced PNPT1 expression (*n*=3). ∗*P* < 0.05, ∗∗∗*P* < 0.001.

Finally, we conducted *in vivo* experiments to manipulate PNPT1 expression specifically in the livers of mice using adeno-associated virus 8 (AAV8) vectors (Figure 8A and Figure S7A). Knockdown of PNPT1 markedly increased hepatic TG, TC, and lipid accumulation (Figures 8B and S7B). Additionally, the expression of inflammatory factors (TNF-α, IL-6, IL-1β) was promoted by downregulated PNPT1 in mouse liver tissue (Figure 8C), whereas PNPT1 overexpression reduced the expression of these pro-inflammatory factors (Figure S7C). It also worsened liver fibrosis and caused mitochondrial swelling (Figures 8F–H and S7F-H). Conversely, overexpression of PNPT1 showed opposite effects. Therefore, modulating PNPT1 expression significantly impacts MAFLD progression *in vivo*.

## 4 Discussion

MAFLD is a metabolic disorder that is rapidly increasing in global prevalence [[50], [51], [52], [53]]. It is associated with heightened risks of obesity, diabetes, and cardiovascular diseases [54,55]. Despite extensive research efforts, the full understanding of MAFLD pathogenesis remains elusive. Current studies highlight mitochondrial dysfunction and cellular apoptosis as critical elements in the progression of MAFLD [56,57]. Therefore, identifying key molecules that regulate these pathological processes is essential for unraveling the pathogenesis of MAFLD. PNPT1 is an important enzyme involved in RNA metabolism and intracellular RNA degradation [58,59]. While its cellular functions have been partially characterized, the specific role of PNPT1 in MAFLD has been unclear.

In our research, we initially observed that PNPT1 expression was notably downregulated in liver tissues from MAFLD patients and in high-fat diet-induced mouse models of the disease. This discovery pointed to PNPT1's possible involvement in MAFLD development. Subsequent functional experiments using cellular models have confirmed that PNPT1 directly influences hepatic lipid synthesis and accumulation and affects apoptosis and mitochondrial stability in hepatocytes. Our findings demonstrate that enhanced PNPT1 expression ameliorates the pathological features of MAFLD hepatocytes, underscoring its significant role as a regulator of key pathological processes in MAFLD.

Further investigations revealed a notable subcellular redistribution of PNPT1 from mitochondria to cytoplasm under high lipid conditions. This shift not only diminishes capacity of PNPT1 to protect mitochondria but also enables cytoplasmic PNPT1 to interact with and degrade Mcl-1 mRNA. This interaction disrupts mitochondrial integrity and enhances the release of PNPT1 into the cytoplasm, thereby establishing a positive feedback loop that impacts both apoptosis and mitochondrial stability. Through structural analysis and vector construction, we identified sequence-specific interactions between the KH and S1 domains of PNPT1 and Mcl-1 mRNA. Moreover, we recognized PPARα as an upstream transcription factor of PNPT1, whose expression is downregulated under MAFLD and FFA conditions, leading to reduced PNPT1 transcription. Our *in vivo* experiments further substantiated that modulating PNPT1 expression significantly influences the progression of MAFLD pathology. Therefore, our study not only advances the understanding of the molecular dynamics in MAFLD but also opens new avenues for therapeutic interventions. PNPT1's involvement in regulating lipid metabolism, apoptosis, mitochondrial permeability, and Mcl-1 expression presents novel targets for therapeutic strategies aimed at managing or reversing MAFLD.

However, the precise modulation of PNPT1 and its broader implications in translational medicine require further exploration. While our research has shown that PNPT1 expression is notably downregulated in both MAFLD and MASH, our primary focus has been on understanding the mechanisms within MAFLD. The observed lower expression of PNPT1 in MASH suggests that it may play an even more critical role in the progression from MAFLD to more severe hepatic conditions. However, the specific role of PNPT1 in MASH, as well as its broader implications, requires further investigation, which we plan to explore in future studies. Moreover, we acknowledge that the sample size in the current study is a limitation, which may affect the generalizability of our findings. Despite this, our results provide valuable insights into the role of PNPT1 in MAFLD. Future research will aim to expand the sample size to validate and extend these findings.

## CRediT authorship contribution statement

**Canghai Guan:** Writing – original draft, Validation, Supervision, Methodology, Investigation, Data curation, Conceptualization. **Xinlei Zou:** Visualization, Software, Methodology, Data curation. **Chengru Yang:** Writing – original draft, Visualization, Validation, Methodology, Data curation. **Wujiang Shi:** Validation, Project administration, Data curation. **Jianjun Gao:** Software, Project administration, Investigation, Formal analysis, Data curation. **Yifei Ge:** Software, Methodology, Investigation. **Zhaoqiang Xu:** Project administration, Data curation. **Shaowu Bi:** Visualization, Resources. **Xiangyu Zhong:** Writing – review & editing, Supervision, Funding acquisition.

## Declaration of competing interest

The authors declare that they have no known competing financial interests or personal relationships that could have appeared to influence the work reported in this paper.

## Data Availability

Data will be made available on request.
