# Supplementary material for: Polyribonucleotide nucleotidyltransferase 1 participates in metabolic-associated fatty liver disease pathogenesis by affecting lipid metabolism and mitochondrial homeostasis
Source: Mol Metab. 2024 Aug 31;89:102022. doi: 10.1016/j.molmet.2024.102022 (PMC11414560; doi:10.1016/j.molmet.2024.102022)
Supplement: Multimedia component 1 [file mmc1.doc]

**
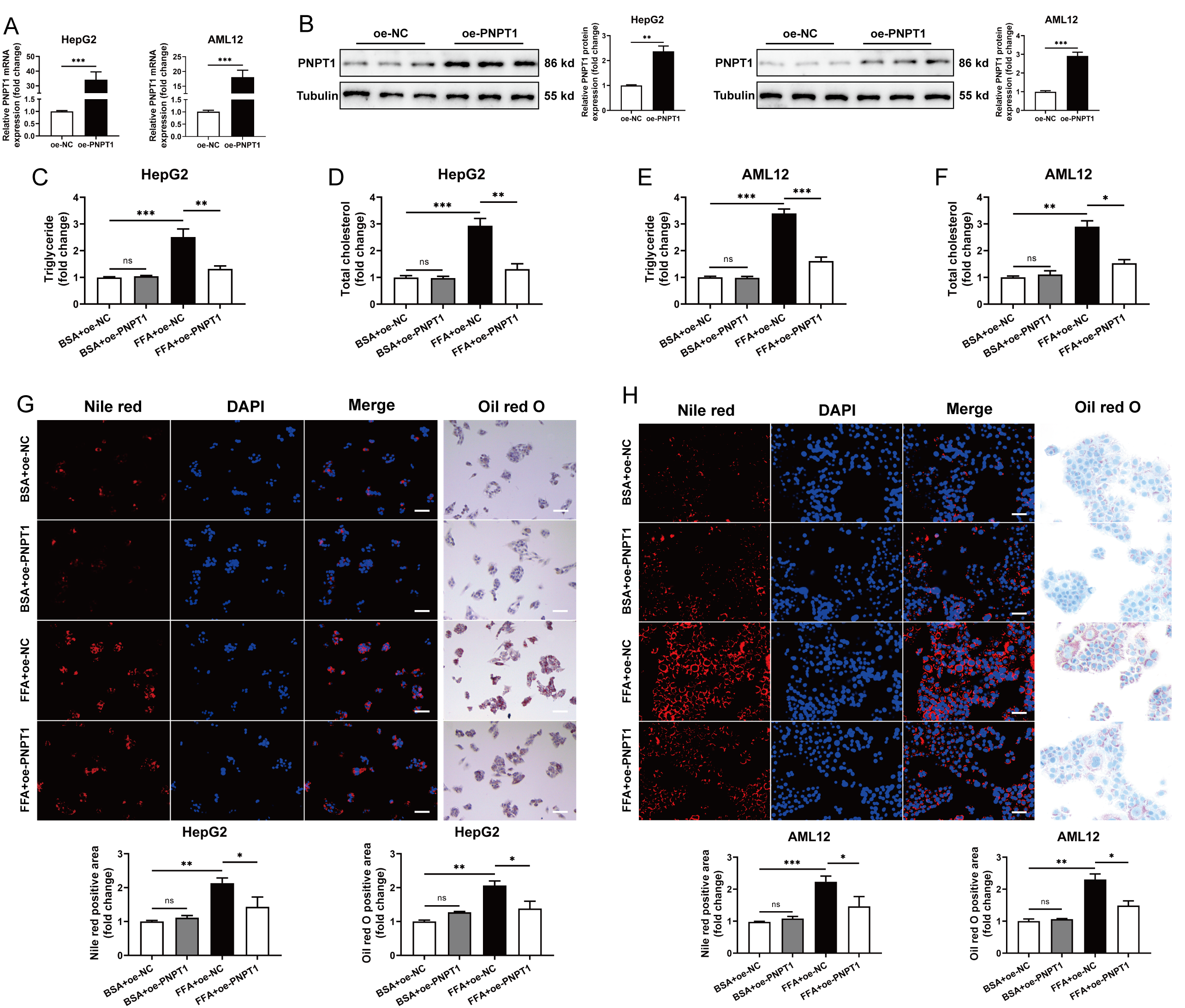
**

**Fig. S1: Influence of PNPT1 on lipid droplet formation and lipid levels in liver cells.** (A, B) Efficacy of PNPT1 overexpression verified by qRT-PCR and western blot in HepG2 and AML12 cells (*n*=5). (C-F) TG and TC assays indicating alterations in lipid content with PNPT1 modulation in HepG2 and AML12 cells (*n*=5). (G, H) Microscopic analysis of lipid droplets via Nile red and Oil red O staining, quantifying the impact of PNPT1 overexpression on lipid storage in FFA-treated liver cells (*n*=5). **P*<0.05, ***P*<0.01, ****P*<0.001.


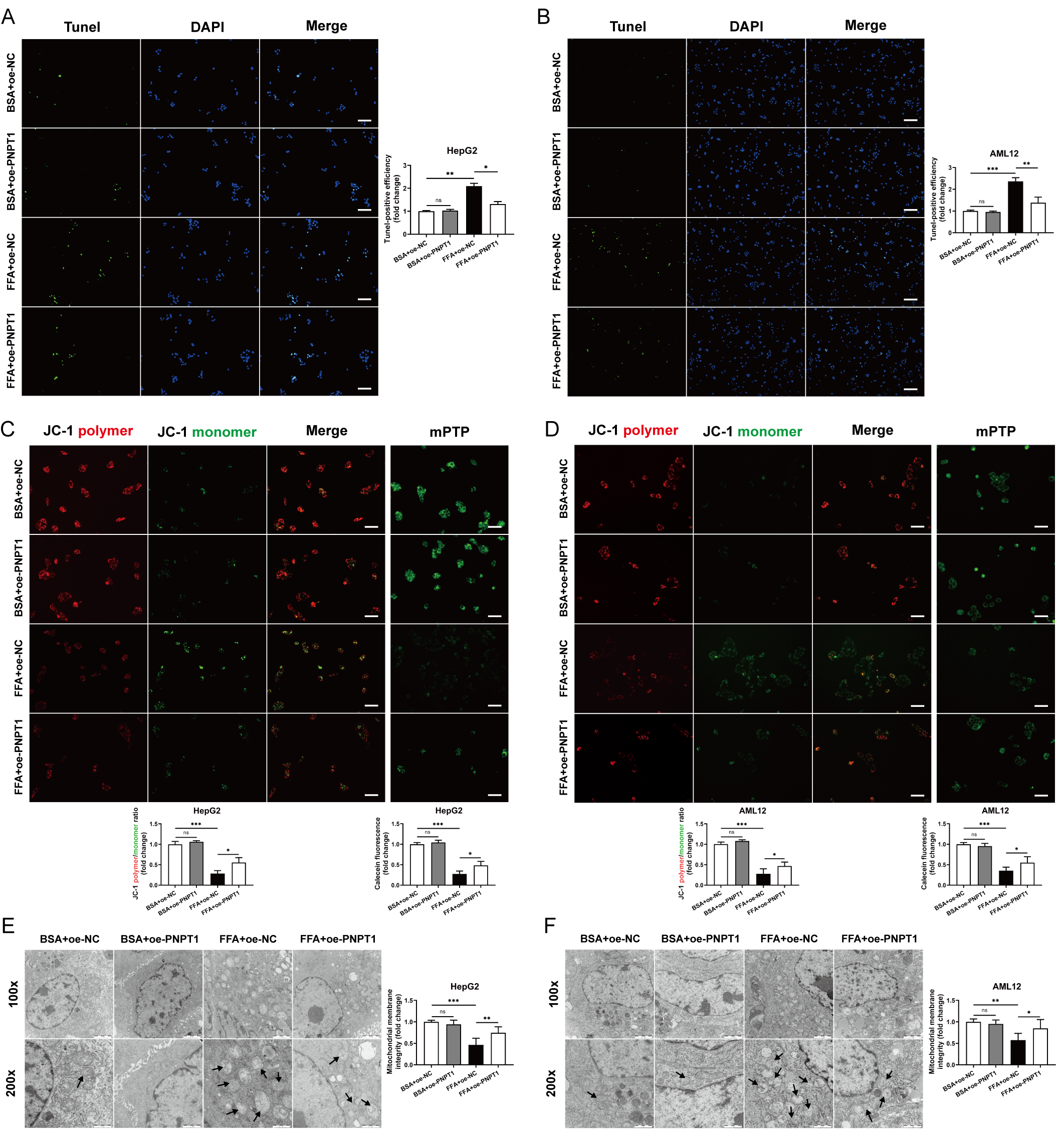


**Fig. S2: Impact of PNPT1 on apoptosis and mitochondrial integrity in** HepG2 and AML12 **cells.** (A, B) Tunel assays indicating a significant decrease in apoptosis rates in FFA-induced HepG2 and AML12 cells following PNPT1 overexpression (*n*=5). (C, D) JC-1 staining and mPTP assays showing changes in mitochondrial membrane potential and decreased permeability pore opening in cells treated with FFA and PNPT1-upregulated cells (*n*=5). (E, F) Electron microscopy images illustrating mitochondrial swelling and deformation in FFA-induced cells after overexpressing PNPT1 (*n*=3). **P*<0.05, ***P*<0.01, ****P*<0.001.


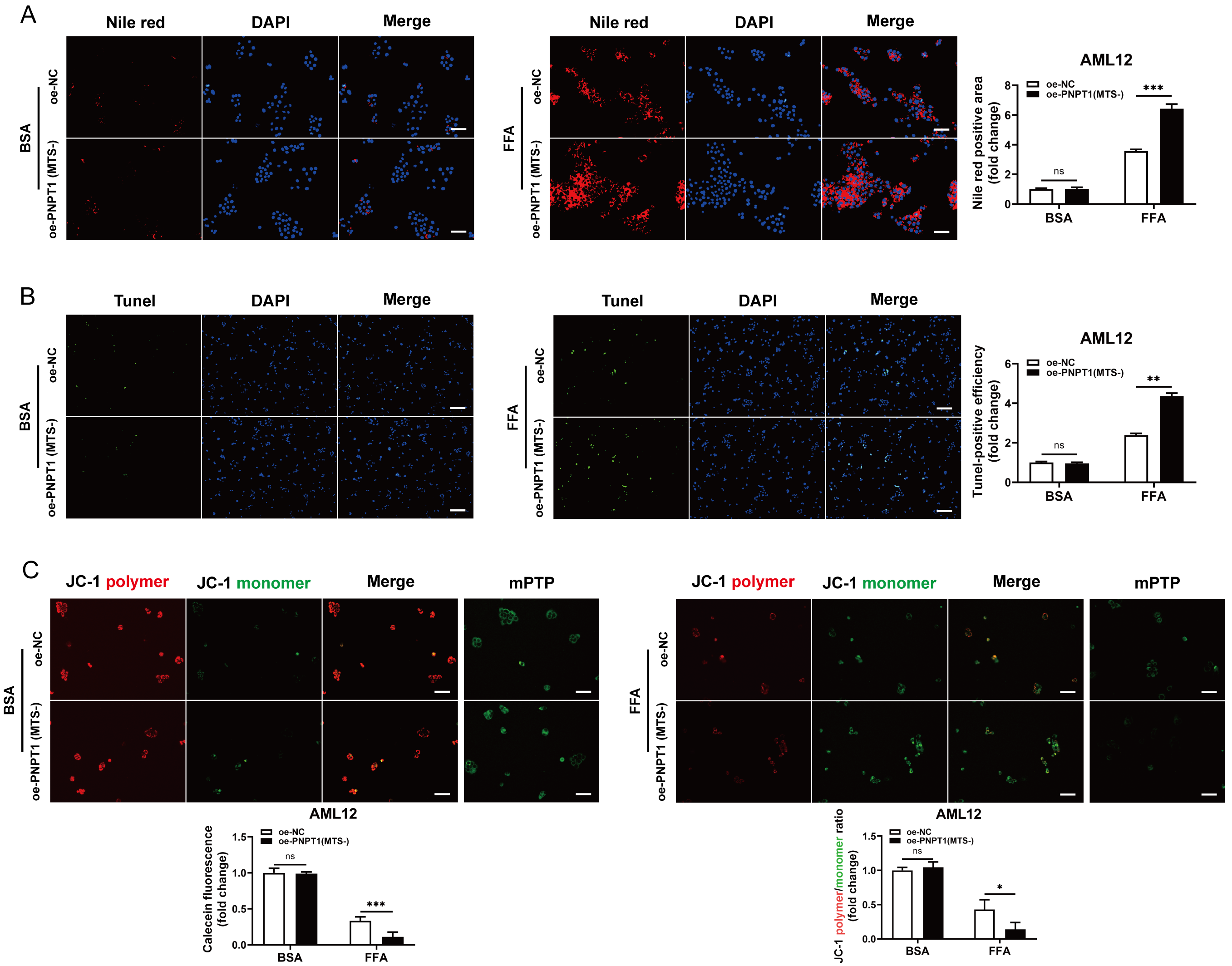


**Fig. S3: Effects of PNPT1 subcellular redistribution on lipid accumulation, apoptosis, and mitochondrial function in AML12 cells.** (A) Nile red staining showing differential lipid accumulation in AML12 cells after FFA treatment. Enhanced lipid accumulation are observed in cells overexpressing MTS-truncated PNPT1 (oe-PNPT1(MTS-)) compared to full-length PNPT1 (oe-PNPT1) and control (*n*=5). (B) Tunel assays indicating increased apoptosis in FFA-induced AML12 cells with cytoplasmic accumulation of PNPT1 due to MTS truncation, compared to cells with mitochondrial PNPT1 enrichment (*n*=5). (C) JC-1 staining and mPTP assays showed that oe-PNPT1(MTS-) promoted the loss of mitochondrial membrane potential and enhanced the opening of mPTP in AML12 (FFA-treated) (*n*=5). **P*<0.05, ***P*<0.01.


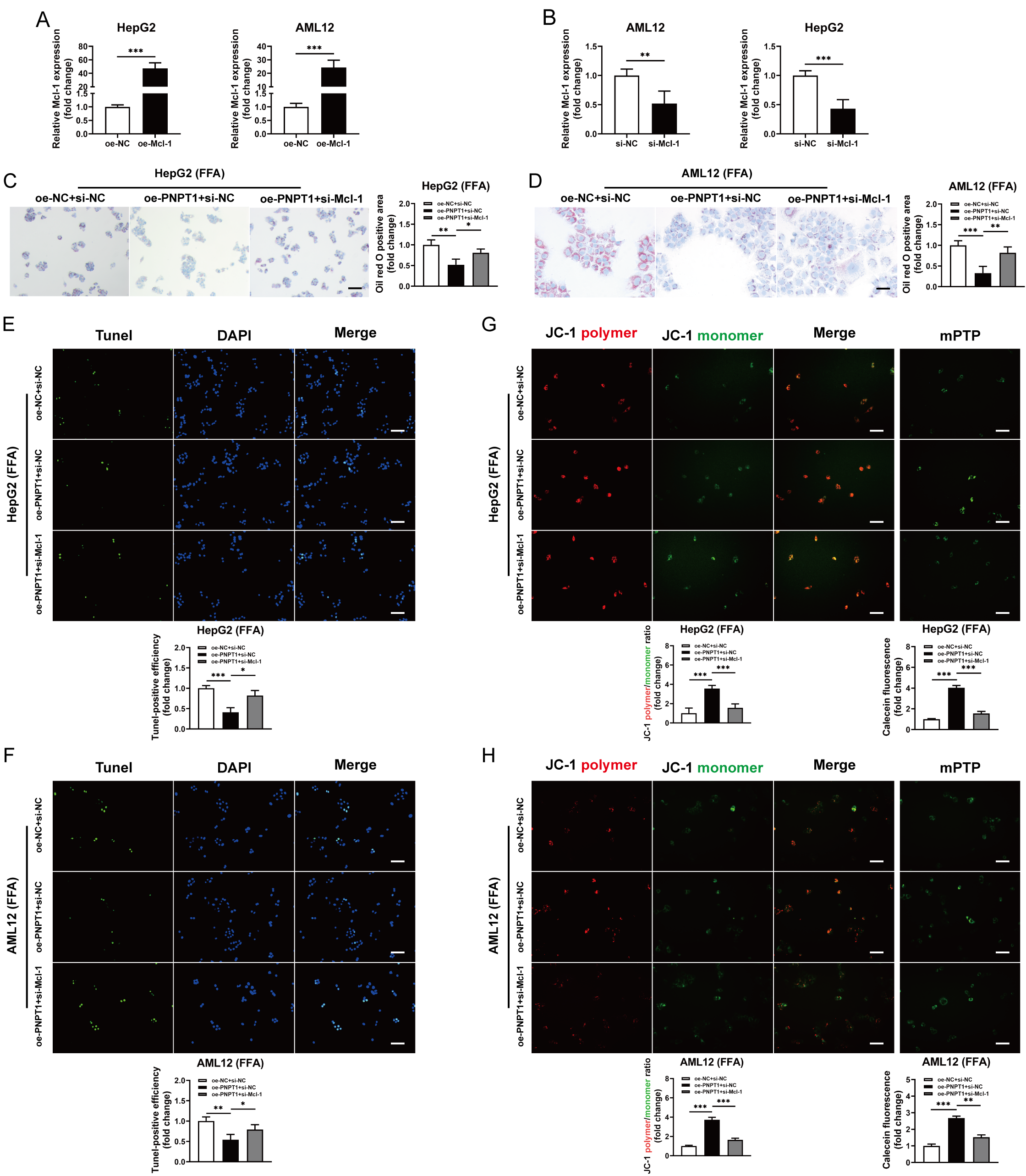


**Fig. S4: Effects of overexpression of PNPT1 and silencing of Mcl-1 on lipid metabolism, mitochondrial stability and apoptosis of hepatocytes.** (A) Efficacy of Mcl-1 overexpression verified by qRT-PCR in HepG2 and AML12 cells. (B) Efficacy of Mcl-1 silencing verified by qRT-PCR in HepG2 and AML12 cells. (C, D) The Oil red O staining results showed that the relief effect of overexpression of PNPT1 on hepatocyte lipid accumulation was up-regulated by low Mcl-1 (*n*=6). (E, F) Tunel assays showed that the inhibitory effect of overexpression of PNPT1 on hepatocyte apoptosis was reversed by silencing Mcl-1. (*n*=6). (G, H) JC-1 and mPTP staining in FFA-treated liver cells demonstrating the reversal of mitochondrial protective effects by the Mcl-1 silencing (*n*=6). **P*<0.05, ***P*<0.01, ****P*<0.001.


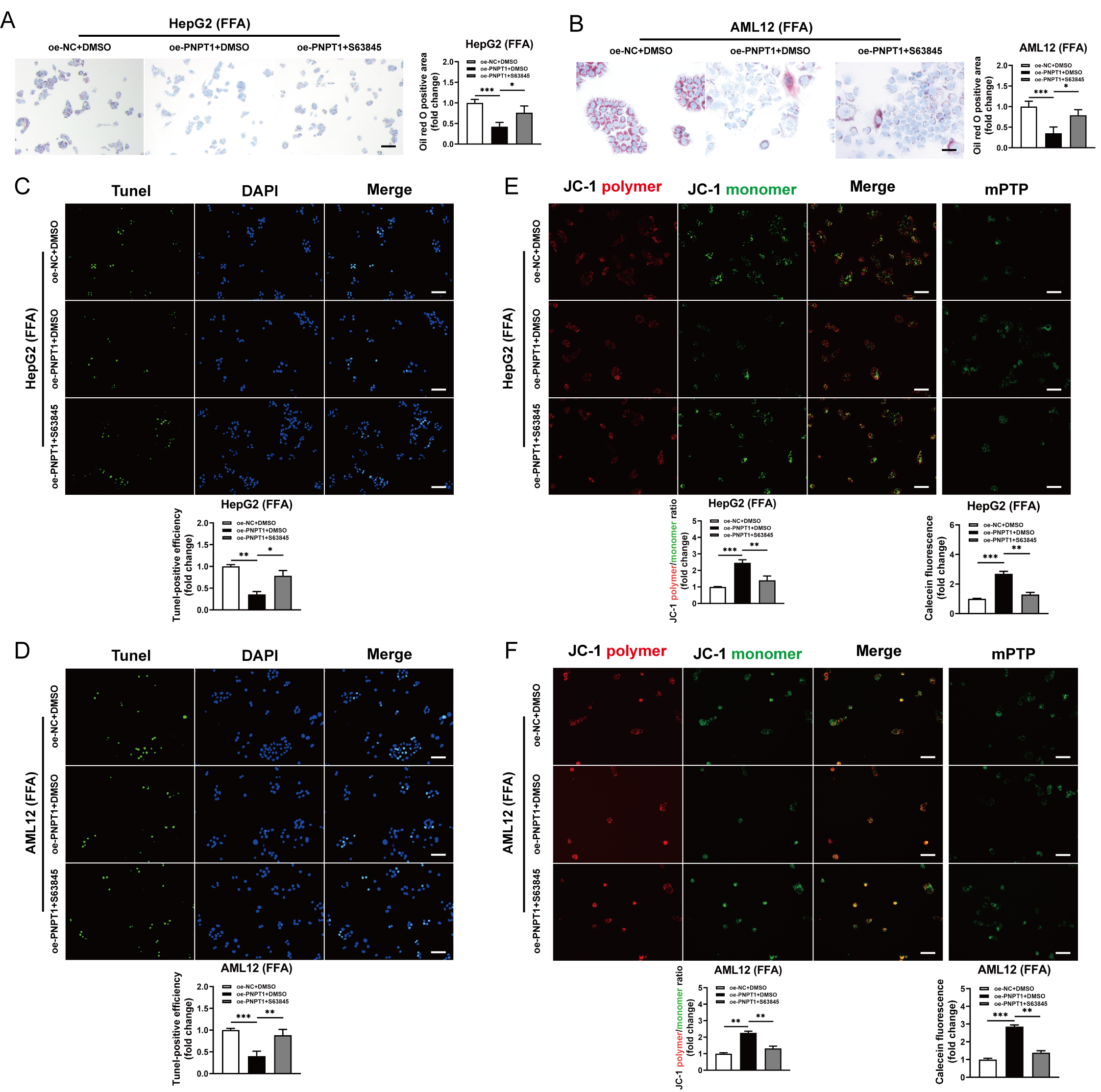


**Fig. S5: Lipid metabolism, mitochondrial stability and apoptotic response in liver cells with PNPT1 and Mcl-1 inhibitor.** (A, B) The Oil red O staining of FFA-induced HepG2 and AML12 cells demonstrates lipid accumulation following PNPT1 overexpression and Mcl-1 inhibitor (S63845) (*n*=5). (C, D) Tunel staining reveals apoptotic cell counts in FFA-treated liver cells with altered PNPT1 expression and subsequent Mcl-1 inhibitor (*n*=5). (E, F) JC-1 staining depicts mitochondrial membrane potential changes, and concurrent mPTP assays results show pore opening in cells treated with FFA, PNPT1 overexpression, and Mcl-1 inhibitor (*n*=5). **P*<0.05, ***P*<0.01, ****P*<0.001.


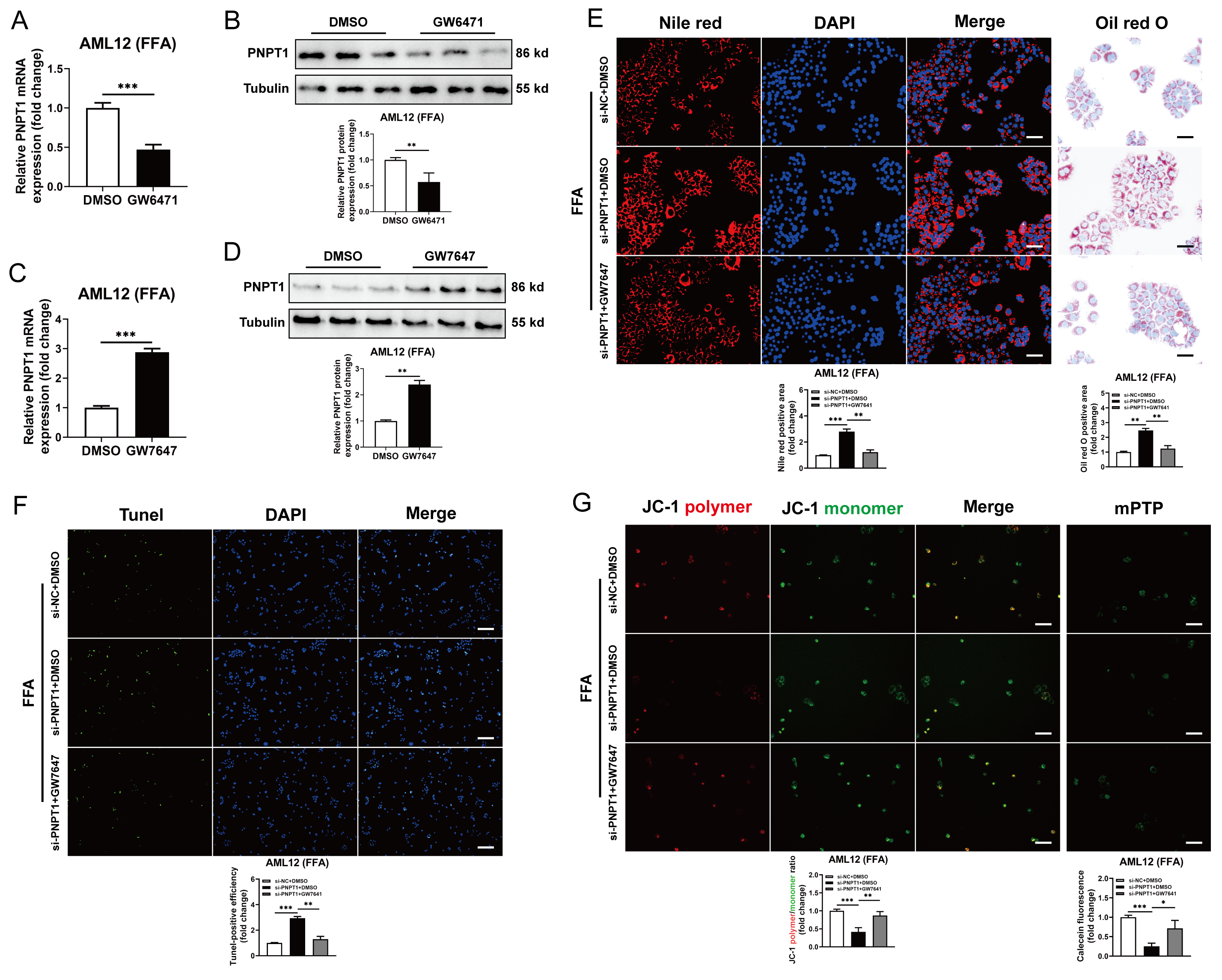


**Fig. S6: Modulation of lipid metabolism and mitochondrial function by PPARα in AML12 Cells.** (A-D) qRT-PCR and western blot analysis showing the effect of GW6471 and GW7647 on PNPT1 expression in AML12 cells (*n*=5). (E-G) Rescue experiments using GW7647 in AML12 cells after FFA treatment reveal that PNPT1 knockdown effects on lipid accumulation, apoptosis, and mitochondrial permeability are mitigated by the PPARα agonist (*n*=5). **P*<0.05, ***P*<0.01, ****P*<0.001.


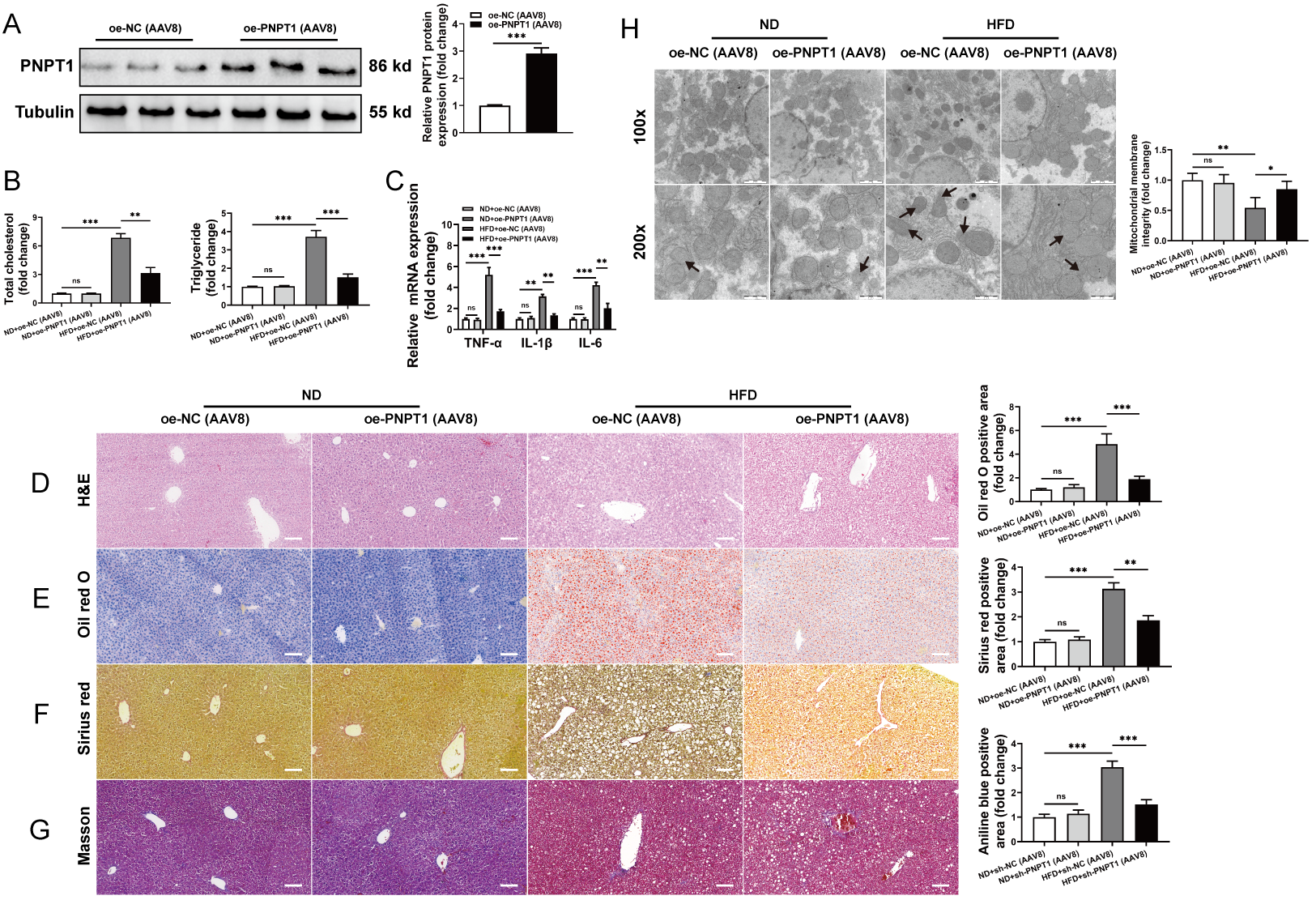


**Fig. S7: PNPT1 overexpression alleviates MAFLD features *in vivo*.** (A) Western blot showing PNPT1 overexpression in mouse livers using AAV8 vectors (*n*=6). (B) Hepatic TG and TC are reduced in mice with PNPT1 overexpression, as shown by biochemical analysis (*n*=6). (C) In PNPT1-upregulated mouse liver, the expression of pro-inflammatory factors (TNF-α, IL-6, IL-1β) was significantly decreased (*n*=6) (D-G) Histological analysis (H&E, Oil red O, Sirius red, and Masson's trichrome) demonstrating decreased lipid deposition and fibrosis in livers of mice overexpressing PNPT1 (*n*=6). (H) Electron microscopy images of liver tissues exhibit normalized mitochondrial morphology in response to PNPT1 overexpression (*n*=3). ***P*<0.01, ****P*<0.001.
