# Supplementary material for: Polyribonucleotide nucleotidyltransferase 1 participates in metabolic-associated fatty liver disease pathogenesis by affecting lipid metabolism and mitochondrial homeostasis
Source: Mol Metab. 2024 Aug 31;89:102022. doi: 10.1016/j.molmet.2024.102022 (PMC11414560; doi:10.1016/j.molmet.2024.102022)
Supplement: Multimedia component 2 [file mmc2.doc]

***Supplementary Material***

**Supplementary Table 1 Clinicopathological characteristics of participants with normal and MAFLD/MASH** patients.

| **Characteristics** | **Normal**  **(n = 8)** | **MAFLD**  **(n = 6)** | **MASH**  **(n = 6)** | ***P* value**  **(MAFLD vs Normal)** | ***P* value**  **(MASH vs Normal)** | ***P* value**  **(MASH vs MAFLD)** |
| --- | --- | --- | --- | --- | --- | --- |
| Sex | Male | Male | Male |  |  |  |
| Age (years) | 54.00 ± 4.75 | 48.33 ± 11.86 | 49.57 ± 10.56 | 0.2392 | 0.3031 | 0.8458 |
| BMI (kg/m^2^) | 21.45 ± 3.28 | 25.78 ± 1.94 | 29.21 ± 4.27 | 0.0142 | 0.0016 | 0.0982 |
| ALT (U/L) | 14.13 ± 6.64 | 37.33 ± 13.87 | 40.43 ± 15.38 | 0.0013 | 0.0007 | 0.7125 |
| AST (U/L) | 19.13 ± 7.18 | 30.17 ± 8.80 | 51.71 ± 21.49 | 0.0237 | 0.0014 | 0.0431 |
| TC (mmol/L) | 4.06 ± 0.91 | 5.31 ± 1.01 | 4.95 ± 0.98 | 0.0314 | 0.0903 | 0.5240 |
| TG (mmol/L) | 1.05 ± 0.33 | 1.83 ± 0.47 | 1.69 ± 0.39 | 0.0034 | 0.0046 | 0.5537 |
| Glucose (mmol/L) | 5.16 ± 0.93 | 6.95 ± 1.54 | 6.94 ± 1.65 | 0.0191 | 0.0214 | 0.9944 |
| PNPT1 expression (mRNA) | 1.00 ± 0.09 | 0.52 ± 0.16 | 0.25 ± 0.19 | 0.0000 | 0.0000 | 0.0189 |
| PNPT1 expression (protein) | 1.00 ± 0.12 | 0.65 ± 0.17 | 0.47 ± 0.15 | 0.0009 | 0.0000 | 0.0700 |

**Supplementary Table 2 Primer sequences for qRT-PCR and siRNAs sequences.**

| **Nucleic acids** | **Sequences** |
| --- | --- |
| GAPDH (human) | F: 5’-GAAGGTGAAGGTCGGAGT-3’ |
|  | R: 5’-GAAGATGGTGATGGGATTTC-3’ |
| β-actin (mouse) | F: 5’-GGCTGTATTCCCCTCCATCG-3’ |
|  | R: 5’-CCAGTTGGTAACAATGCCATGT-3’ |
| PNPT1 (human) | F: 5’-TGCAGTAATGGTCACAGCGG-3’ |
|  | R: 5’-AGTAGCCAGCTGGAAAGAGC-3’ |
| PNPT1 (mouse) | F: 5’-CCTGTTGGGGCAGTACGAAT-3’ |
|  | R: 5’-CCTGTTGGGGCAGTACGAAT-3’ |
| Mcl-1 (human) | F: 5’-GGGCGACTTTTGGCCACC-3’ |
|  | R: 5’-GCTAGGTTGCTAGGGTGCAA-3’ |
| Bcl-xl (human) | F: 5’-CCTAAGGCGGATTTGAATAATCTT-3’ |
|  | R: 5’-AGGTAAGTGGCCATCCAAGC-3’ |
| PPARα (human) | F: 5’-CCTGTCTGCTCTGTGGACTC-3’ |
|  | R: 5’-TGAAAGCGTGTCCGTGATGA-3’ |
| PPARα (mouse) | F: 5’-TGCCTTCCCTGTGAACTGAC-3’ |
|  | R: 5’-TGGGGAGAGAGGACAGATGG-3’ |
| E1 | F: 5’-AGGCAATACTGCCCAAATGA-3’ |
|  | R: 5’-ACCTACTTGCCACAATTGAACA-3’ |
| E2 | F: 5’-AGGAGACGCAAAAACTGCTT-3’ |
|  | R: 5’-TGACACCTTGCTTTCATGAGC-3’ |
| E3 | F: 5’-AGTTCGACCAAAAGGGACAGA-3’ |
|  | R: 5’-CCTTCTCCGCCCTGGTTTTT-3’ |
| TNF-α (mouse) | F: 5’-CCTCTCTCTAATCAGCCCTCTG-3’ |
|  | R: 5’-GAGGACCTGGGAGTAGATGAG-3’ |
| IL-1β (mouse) | F: 5’-ATGATGGCTTATTACAGTGGCAA-3’ |
|  | R: 5’-GTCGGAGATTCGTAGCTGGA-3’ |
| IL-6 (mouse) | F: 5’- AAATTCGGTACATCCTCGACGG3’ |
|  | R: 5’-GGAAGGTTCAGGTTGTTTTCTGC-3’ |
| si-NC (human) | 5’-UUCUCCGAACGUGUCACGUTT-3’ |
|  | 5’-ACGUGACACGUUCGGAGAATT-3’ |
| si-PNPT1 (human) | 5’-CGCCAGAGAUUGUGAAAUAUATT-3’ |
|  | 5’-UAUAUUUCACAAUCUCUGGCGTT-3’ |
| si-NC (mouse) | 5’-UUCUCCGAACGUGUCACGUTT-3’ |
|  | 5’-ACGUGACACGUUCGGAGAATT-3’ |
| si-PNPT1 (mouse) | 5’-AUAUCAGGUUCAUUGAUGCCATT-3’ |
|  | 5’-UGGCAUCAGAACCUGAUAUTT-3’ |
| si-Mcl-1 (human) | 5’-GGAUAGUAGUGUUGUAAGAGA-3’ |
|  | 5’-UCUUACAACACUACUAUCCAG-3’ |
| si-Mcl-1 (mouse) | 5’-GGCUAGUCACAAAGCUCAAUA-3’ |
|  | 5’-UUGAGCUUUGUGACUAGCCUG-3’ |

**Supplementary Table 3 A**ntibodies used for western bolt

| **Antibodies** | **Vendor** | **Catalog** | **Dilution ratio** |
| --- | --- | --- | --- |
| PNPT1 | Proteintech | 14487-1-AP | WB: 1:4000 |
|  |  |  | IF: 1:200 |
|  |  |  | IP: 4.0 ug/ml |
| Tubulin | Abcam | ab7291 | WB: 1:5000 |
| COX IV | Abcam | ab16056 | WB: 1:5000 |
| Bcl-2 | Proteintech | 12789-1-AP | WB: 1:2000 |
| Bcl-xl | Proteintech | 26967-1-AP | WB: 1:2000 |
| Mcl-1 | Proteintech | 16225-1-AP | WB: 1:2000 |
| HA tag | Abcam | ab9110 | IP: 2.0 ug/ml |
| PPARα | Abcam | ab227074 | WB: 1:1000 |
|  |  |  | IP: 5.0 ug/ml |
